# Comparative analysis of wild-type and chloroplast MCU-deficient plants reveals multiple consequences of chloroplast calcium handling under drought stress

**DOI:** 10.3389/fpls.2023.1228060

**Published:** 2023-08-25

**Authors:** Francesca Corti, Margherita Festa, Frank Stein, Piergiorgio Stevanato, Jitka Siroka, Lorella Navazio, Ute C. Vothknecht, Alessandro Alboresi, Ondřej Novák, Elide Formentin, Ildikò Szabò

**Affiliations:** ^1^ Department of Biology, University of Padua, Padua, Italy; ^2^ European Molecular Biology Laboratory, Heidelberg, Germany; ^3^ Department of Agronomy, Food, Natural Resources, Animals and Environment, University of Padua, Padua, Italy; ^4^ Laboratory of Growth Regulators, Institute of Experimental Botany of the Czech Academy of Sciences & Palacký University, Olomouc, Czechia; ^5^ Plant Cell Biology, Institute of Cellular and Molecular Botany, University of Bonn, Bonn, Germany

**Keywords:** chloroplast, calcium channel, calcium sensor, drought stress, comparative proteomics

## Abstract

**Introduction:**

Chloroplast calcium homeostasis plays an important role in modulating the response of plants to abiotic and biotic stresses. One of the greatest challenges is to understand how chloroplast calcium-permeable pathways and sensors are regulated in a concerted manner to translate specific information into a calcium signature and to elucidate the downstream effects of specific chloroplast calcium dynamics. One of the six homologs of the mitochondrial calcium uniporter (MCU) was found to be located in chloroplasts in the leaves and to crucially contribute to drought- and oxidative stress-triggered uptake of calcium into this organelle.

**Methods:**

In the present study we integrated comparative proteomic analysis with biochemical, genetic, cellular, ionomic and hormone analysis in order to gain an insight into how chloroplast calcium channels are integrated into signaling circuits under watered condition and under drought stress.

**Results:**

Altogether, our results indicate for the first time a link between chloroplast calcium channels and hormone levels, showing an enhanced ABA level in the cmcu mutant already in well-watered condition. Furthermore, we show that the lack of cMCU results in an upregulation of the calcium sensor CAS and of enzymes of chlorophyll synthesis, which are also involved in retrograde signaling upon drought stress, in two independent KO lines generated in Col-0 and Col-4 ecotypes.

**Conclusions:**

These observations point to chloroplasts as important signaling hubs linked to their calcium dynamics. Our results obtained in the model plant *Arabidopsis thaliana* are discussed also in light of our limited knowledge regarding organellar calcium signaling in crops and raise the possibility of an involvement of such signaling in response to drought stress also in crops.

## 1 Introduction

Plant bioenergetic organelles, namely mitochondria and chloroplasts, display specific calcium (Ca^2+^) signals upon abiotic and biotic stresses (McAinsh and Pittman, 2009; Rizzuto et al., 2012; Rocha and Vothknecht, 2012; Nomura and Shiina, 2014; Kmiecik et al., 2016; Wagner et al., 2016). Cytosolic free Ca^2+^ concentration transiently raises under osmotic stress, high salt, cold as well as oxidative stress, triggering Ca^2+^ uptake into chloroplasts. In turn, cytosolic Ca^2+^ signatures (Nomura et al., 2012; Loro et al., 2016; Sello et al., 2016) can be fine-tuned by sequestration of Ca^2+^ in intracellular organelles (Costa et al., 2018), as proven by measuring cytoplasmic and chloroplast Ca^2+^ dynamics using genetically encoded Ca^2+^ indicators (GECIs) (Loro et al., 2016; Sello et al., 2016; Sello et al., 2018). This approach revealed stimulus-specific, regulated changes in Ca^2+^ level (Sai and Johnson, 2002; Loro et al., 2016; Teardo et al., 2019) in the chloroplast (for review see e.g. Costa et al., 2018; Navazio et al., 2020).

Until recently, the molecular identity of most chloroplast channels has remained unclear (Carraretto et al., 2016; Navazio et al., 2020). During the last few years, different channels/transporters able to transport Ca^2+^ or modulate Ca^2+^ entry into chloroplasts have been described, including the bivalent cation transporter BICAT2, able to transport Mn^2+^ as well and import cytosolic Ca^2+^ during the light phase of photosynthesis (Schneider et al., 2016; Frank et al., 2019), plastid-located cationic glutamate receptors GLR3.4 (Teardo et al., 2011) and GLR3.5 (Teardo et al., 2015) as well as two channels (PEC1/2) with undefined selectivity whose deletion largely decreases chloroplast Ca^2+^ release (Völkner et al., 2021).

We have recently shown in *Arabidopsis thaliana* that one of the six homologs of the Ca^2+^-selective mammalian mitochondrial uniporter (MCU) (De Stefani et al., 2011; Stael et al., 2012), named cMCU (for chloroplast MCU, At5g66650) mediates Ca^2+^ uptake into mature chloroplasts in the leaves upon osmotic and oxidative stress. These differences translated to a different activation level of MAPK3/6 by a still unknown mechanism, leading to altered activation of ERF6 and of guard-cell specific MYB60 transcription factors, impinging on short-term signaling that links chloroplast Ca^2+^ dynamics to stomatal closure. Interestingly, we also provided evidence that *Arabidopsis* plants lacking cMCU (*cmcu*) have constitutively partially closed stomata and are more resistant to long-term drought with respect to wild type (WT) plants. At the same time, these mutant plants maintained photosynthetic activity even after 18 days of water deprivation, in contrast to WT plants (Teardo et al., 2019). The molecular mechanisms underlying this relevant phenotype are unknown.

In order to address this question, we performed comparative transcript analysis as well as proteomic, ionomic and hormonal analysis of WT and *cmcu* leaves of plants grown under watered and drought conditions. We aimed to clarify the mechanism of drought tolerance and which proteins may play a role in cMCU-mediated pathways. Such understanding might increase our ability to improve stress resistance in crops to achieve agricultural sustainability and food security for an ever-growing world population (Zhu, 2016) in a scenario of climate changes.

## 2 Materials and methods

### 2.1 Plant materials and growth conditions

*Arabidopsis thaliana* plants were grown in short day photoperiod, with 12 h light (red and blue led light, about 200 μmol photons m^-2^ s^-1^) and 12 h dark, a 65-70% relative humidity and a temperature of 22°C in the light, 20°C in the darkness. Seeds were sown on autoclaved soil and grown with regular watering. For drought stress treatment, 4 weeks old plants were subjected to water withdrawal for 7 to 15 days, then sampled for subsequent experiments. Leaf samples were used for RNA or protein extraction, and adult leaves of similar dimension and positions in the rosette were taken from each plant. Two independent T-DNA insertion lines for AtMCU6 named also cMCU (At5g66650 locus), namely *cmcu-1* (SK16605, from Saskatoon Arabidopsis T-DNA mutant collection) and *cmcu-2* (SALK_031408 from the SALK collection), were used as *cMCU* knockouts, with their corresponding wild-type ecotypes Columbia 4 (Col-4, genetic background for *cmcu-1*) and Columbia 0 (Col-0, for *cmcu-2*). All seeds were originally obtained from NASC (Nottingham Arabidopsis Stock Centre). Lack of cMCU in both *cmcu-1* and *cmcu-2* was confirmed as in (Teardo et al., 2019) (not shown), while the expression level of all other MCU isoforms (AtMCU1 [At1g09575], AtMCU2 [At1g57610], (AtMCU3 [At2g23790], AtMCU4 [At4g36820], AtMCU5 [At5g42610]) was comparable between the mutant lines used in this work and their respective WT lines, as assessed by RT-qPCR (**Supplementary Figure 1**). Although *cmcu-1* contains a 4x 35S activation tag at the right border, our proteomic analysis revealed that the expression level for example of the related MCU5 (Ruberti et al., 2022) was not different for the *cmcu-1* versus Col-4 (logFC value of -0.0243 was detected, not shown), suggesting that eventual post-transcriptional silencing of related MCUs did not take place in *cmcu-1*.

### 2.2 Proteomics and bioinformatic analysis

Total lysates were prepared from leaf samples by crushing the leaves with a sterile pestle in liquid nitrogen, and subsequently solubilizing proteins with a fresh 50 mM TRIS-HCl, 10% sucrose, 2% SDS, 1 mM EDTA buffer with the addition of Protease Inhibitor Cocktail (P9599, Sigma-Aldrich). The total lysates were centrifuged to remove cellular debris, then treated with Benzonase nuclease (Millipore). Samples were quantified with the Pierce BCA Protein Assay kit (Thermo Scientific) and diluted to 1 µg/µL total proteins. Proteomics experiments on the total protein samples were carried out at the European Molecular Biology Laboratory (EMBL) - Proteomics Core Facility, using mass spectrometry (MS) coupled to isotope labeling of peptides using tandem mass tags (TMTs). Protein extracts isolated from cells or tissues were reduced, alkylated, and then digested. Samples were then labeled with the TMT reagents before sample mixing, fractionation, and cleanup. Labeled samples were analyzed on a high-resolution Orbitrap LC-MS/MS mass spectrometer before data analysis to identify peptides and quantify relative abundance of reporter ions. The samples from the different lines and plants were processed in the same moment and analyzed in the same TMT experiment. The raw output files of IsobarQuant (protein.txt – files) were processed using the R programming language (ISBN 3-900051-07-0). Contaminants were filtered out and only proteins that were quantified with at least two unique peptides were considered for the analysis. 5215 proteins passed the quality control filters. Log2 transformed raw TMT reporter ion intensities (‘signal_sum’ columns) were first cleaned for batch effects using the ‘removeBatchEffects’ function of the limma package (PMID: 25605792) Ritchie et al, (2015) and further normalized using vsn (variance stabilization normalization - PMID: 12169536) Huber et al, (2022). Proteins were tested for differential expression using the limma package. The replicate information was added as a factor in the design matrix given as an argument to the ‘lmFit’ function of limma. A protein was annotated as a hit with a false discovery rate (FDR) smaller than 5% and a fold-change of at least 50%, and as a candidate with a FDR below 20% and a fold-change of at least 20%. Proteins found to be differentially expressed in proteomics were analyzed for Gene Ontology class enrichment using Panther tool, version 17.0 (pantherdb.org) and ShinyGO tool, version 0.76 (http://bioinformatics.sdstate.edu/go/), and also represented in networks using STRING multiple protein search (https://string-db.org).

### 2.3 RNA extraction and RT-qPCR analysis

RNA was extracted from *Arabidopsis* adult leaves using PrimeZOL reagent (Canvax), according to the protocol described in the data sheet. Residual DNA was digested by incubating the RNA solution with Ambion DNase I (RNase-free) (Invitrogen) at 37°C for 30 min, and the enzyme was then inactivated by 10 min incubation at 75°C in the presence of 5 mM EDTA. Subsequently, RNA was precipitated and resuspended in nuclease-free water. DNA degradation and RNA integrity were verified by loading the RNA in an agarose gel electrophoresis, then 2.5 µg of RNA were retrotranscribed with the SuperScript IV Reverse Transcriptase (Invitrogen). Quantitative real time PCR (RT-qPCR) was performed using a CFX384 Touch Real-Time PCR System (BioRad), with standard cycling parameters, preparing the reaction mix with Power SYBR Green Master Mix (ThermoFisher Scientific) and 20 ng cDNA (n=3 biological samples, 3 technical replicates for each sample). Threshold cycle (Ct) values were extrapolated from amplification curves, and melting curves were checked to ensure single-peaked amplification of the target gene. Ct values were averaged across technical replicates, then the ΔCt was calculated for each gene/condition as average Ct (gene) – average Ct (actin). Gene expression was normalized on WT watered samples, by calculating ΔΔCt as ΔCt (gene, condition) - ΔCt (gene, WT W), from which the fold change can be obtained as 2^-ΔΔCt^. Primer sequences used for genotyping and transcript amplification of the cMCU gene and for RT-qPCR analyses used in this study are provided in **Supplementary Table 1**.

### 2.4 Leaf photoinhibition assay

*Arabidopsis* plants were grown on soil in control conditions (see above) until about 30 days of age, then adult leaves of similar dimension and positions in the rosette were cut from each plant and positioned on a tray with multiple layers of wet filter paper, in order to prevent leaf desiccation. The tray was positioned under a high light intensity lamp (white led light, 1500 μmol photons m^-2^ s^-1^) and kept wet for the whole duration of the experiment. For the first 8 hours, the leaves were removed from under the lamp every hour and kept in the dark for 20 minutes, then leaf PSII quantum efficiency was assessed through chlorophyll fluorescence measurements using a Closed FluorCam FC 800-C setup (Photon System Instrument), using an 800 ms saturating light pulse of about 2700 μmol photons m^-2^ s^-1^. PSII quantum efficiency was calculated as Fv/Fm, where Fm is the maximal leaf fluorescence value in the presence of the saturating pulse, and Fv=(Fm-F_0_) is the difference between the maximal and baseline fluorescence values.

### 2.5 Pigment quantification

Chlorophylls and carotenoids were quantified in fresh adult leaves of *Arabidopsis* WT and *cmcu* plants (n=8). The pigments were extracted from leaves using dimethylformamide (DMF) as solvent, and subsequently quantified by measuring light absorption at 647 and 664 nm for chlorophylls, with the spectrophotometer zeroed at 750 nm, according to (Porra et al., 1989) and at 480 nm for carotenoids, according to (Wellburn, 1994). The equations indicated in the references were used to calculate pigment concentration in µg/mL from absorbance values. Pigment content was then reported as a ratio to the leaves’ fresh weight.

### 2.6 Ionomics

Element content was assessed from pooled *Arabidopsis* rosettes of both regularly watered (n=3 biological samples, 3 technical replicates for each sample) and drought-stressed (n=2, 3 technical replicates for each sample) Col-0 and *cmcu-2* plants. The analysis was performed as described previously (Stevanato et al., 2018). Briefly, leaf samples were treated with concentrated HNO_3_ in a microwave system. The elements concentration was determined by inductively coupled plasma inductively coupled plasma optical emission spectroscopy (ICP-OES) (Ciros Vision EOP, Spectro A. I. GmbH, Germany). Elements were quantified using certified multi-element standards. The element content was normalized on the samples’ dry weight.

### 2.7 Phytohormone quantification

Adult, fully expanded leaves were sampled from *Arabidopsis* plants. To partially compensate for response and biological variability, the phytohormone analysis was based on pooled leaf material: 5 different samples each containing 3 leaves from 3 different plants were tested in 6 technical replicates. The plant hormones were quantified using liquid chromatography tandem mass spectrometry methods as described in (Antoniadi et al., 2015; Široká et al., 2022). For auxin, abscisic acid (ABA) and jasmonate families the samples were prepared using acidic extraction in 1 mol/L formic acid in 10% aqueous methanol and further processed as in (Floková et al., 2014). For the cytokinin family the samples were prepared following protocol in (Antoniadi et al., 2015).

### 2.8 Statistical analysis and graphs

Final data obtained from the several biological replicates in the RT-qPCR, high light, photoinhibition, pigment quantification, ionomics and phytohormone quantification experiments were analyzed to identify statistically significant differences across genotypes and/or conditions, and plotted in suitable graphs for visualization. Graphs and statistical analyses were prepared using GraphPad Prism software, and differences were assessed with t-tests, when dealing with 2 genotypes only (e.g. Col-0 *versus cmcu-2* in ionomics and hormone quantification), or with one-way ANOVA coupled with multiple comparisons-corrected tests, when dealing with more genotypes or conditions, as in the case of RT-qPCR analysis, high light experiments and pigment quantification. For the photoinhibition experiment, a two-way ANOVA was used to analyze time-course measurements, in order to study variability derived from different genotypes and different timepoints. Moreover, a one-way ANOVA coupled with multiple comparisons across genotypes was used when focusing on the 24 hours timepoint.

## 3 Results

### 3.1 Comparative proteomics reveal differentially expressed proteins in cMCU knock-out plants under control conditions

**Figure 1:**
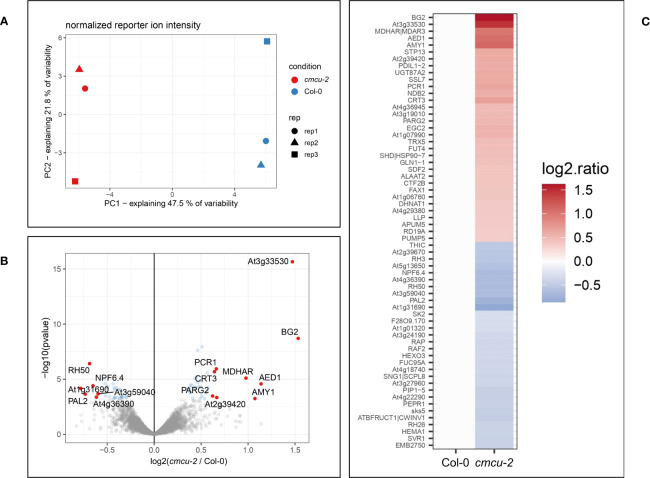
Comparative proteomic analysis of *cmcu-2* plants in non-stressed conditions. **(A)** Principal component analysis of *cmcu-2* and Col-0 WT leaf proteomic samples (n=3). **(B)** Volcano plot showing proteins detected in proteomic analysis, log_2_(FC) values and -log_10_(pvalue) for each protein in *cmcu-2* compared to Col-0 WT. Hit proteins are highlighted in red, while candidates are shown in blue. **(C)** Heatmap of hit and candidate proteins detected by proteomics in *cmcu-2* leaves, showing their log_2_(FC) compared to Col-0 WT.

In order to reveal constitutive differences in the expression of proteins under control, non-stressed conditions between the Col-0 ecotype and the *AtMCU6* (from this point on named *cMCU)* knock-out (KO) line *cmcu-2* in the same background (Teardo et al., 2019), we performed a comparative proteomics analysis, sampling adult leaves from 3 independent 4 weeks-old plants for each genotype. We performed quantitative mass spectrometry experiments using tandem mass tag (TMT) labeling. Normalized reporter ion intensities were tested for differential expression using the limma package (moderate t-test). Proteins were considered “hits” for a specific comparison if the fold change was over 1.5 (logFC > 0.58 or logFC < -0.58). Proteins were moreover considered “candidates” when the proteins had a fold change over 1.2 (logFC > 0.26 or logFC < -0.26). The false discovery rate (FDR) was ≤ 0.05 for proteins of both categories. For global proteome comparison among WT and genetically modified organisms/cells, a value of 1.2-fold change with p value ≤0.05 is routinely used [see e.g (Chakraborty et al., 2018; Liu et al., 2020)]. Setting these threshold values, we identified 63 differentially expressed proteins (DEPs, intended as changes in protein content), 9 of which were considered as upregulated hits, 6 as downregulated hits, 24 as upregulated candidates and 24 as downregulated candidates (**Supplementary Table 2**). Principal component analysis showed that samples from the same genotype grouped together and were clearly distinguishable (**Figure 1A**).

**Figure 2:**
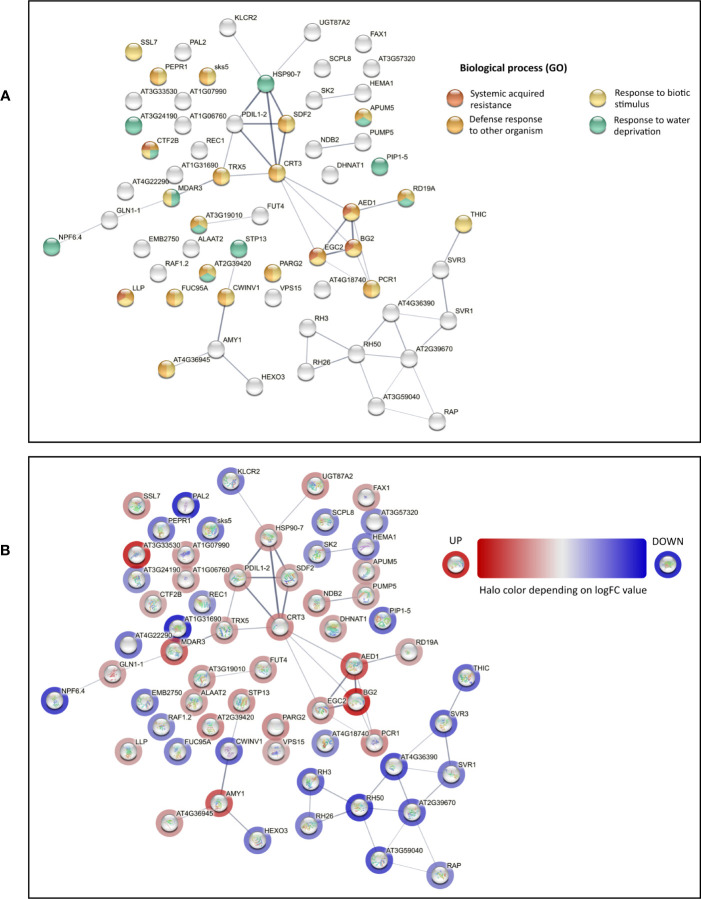
STRING network of differentially expressed proteins (DEPs) in the proteomics analysis of non-stressed *cmcu-2* and Col-0 plants. **(A)** STRING network of DEPs found in proteomics analysis for non-stressed *cmcu-2* leaves. Enrichment analysis was performed with Panther and relevant enriched classes in the Biological Process Ontology were highlighted on the network. **(B)** STRING network for the same DEPs, with halo color indicating the logFC values of DEPs in *cmcu-2* samples.

Proteins found to be differentially expressed in *cmcu-2* are represented in a Volcano plot (**Figure 1B**), a heatmap (**Figure 1C**) and a network using STRING (annotations were updated using Panther and the Gene Ontology database) (**Figure 2**). We used the Panther tool to query Gene Ontology (GO) database, considering the Biological Process ontology: proteins belonging to classes found to be enriched among DEPs in *cmcu-2* are highlighted in different colors in **Figure 2A**. The degree of up- (red) or downregulation (blue) is represented for each DEP in **Figure 2B**. The full list of DEPs found in *cmcu-2* compared to WT Col-0 plants, together with the GO annotation obtained by querying the GO database through the Panther tool and selecting enriched GO classes of particular interest, is reported in **Supplementary Table 2**. The DEPs we identified fall, among others, into the following enriched GO categories, with a FDR value <0.05: response to stress (34 out of 63 proteins), response to water deprivation (11/63), systemic acquired resistance (5/63), defense response to other organism (19/63) and response to biotic stimulus (22/63). In addition, 21 DEPs fall into the category of “response to abiotic stimulus” with and FDR <0.1. The enrichment of Gene Ontology Cellular Component classes found among DEPs (obtained using Panther) is represented in **Table 1**. An enrichment is found for proteins located in the chloroplast and in secretory vesicles and extracellular compartments. Conversely, no enrichment was found for mitochondrial or vacuolar proteins, in support of the chloroplast localization of cMCU in fully expanded leaves (Teardo et al., 2019).

**Table 1:** Enriched GO Cellular Component classes found among differentially expressed proteins (DEPs) in *cmcu-2* non-stressed samples.

GO-term	Cellular component	Protein n. (in 63)	Fold enrichment	FDR
**GO:0048046**	**apoplast**	7	10.32	2.02E-03
**GO:0009507**	**chloroplast**	26	2.30	3.99E-03
**GO:0099503**	**secretory vesicle**	5	13.00	9.86E-03
**GO:0005618**	**cell wall**	7	5.88	2.86E-02
**GO:0005773**	vacuole	7	2.99	3.96E-01
**GO:0005739**	mitochondrion	12	1.22	1.00
**GO:0005783**	endoplasmic reticulum	5	2.02	1.00

Results of Panther enrichment analysis are reported for significantly enriched classes (FDR<0.05, in bold) and some other relevant, non-enriched classes.

Interestingly, the most highly upregulated protein in the *cmcu-2* plants compared to *WT* ones was BG2 (2.9-fold increase), a β-glucosidase that, along with its ER-located homolog BG1, can catalyze one-step hydrolysis of Glucose-conjugated ABA (Xu et al., 2012). The BG2 enzyme is thus able to increase abscisic acid (ABA) levels, independently of the *de novo* synthesis of this plant hormone (Xu et al., 2012).

### 3.2 Characterization of hormone content and ionome in plants lacking cMCU

**Figure 3:**
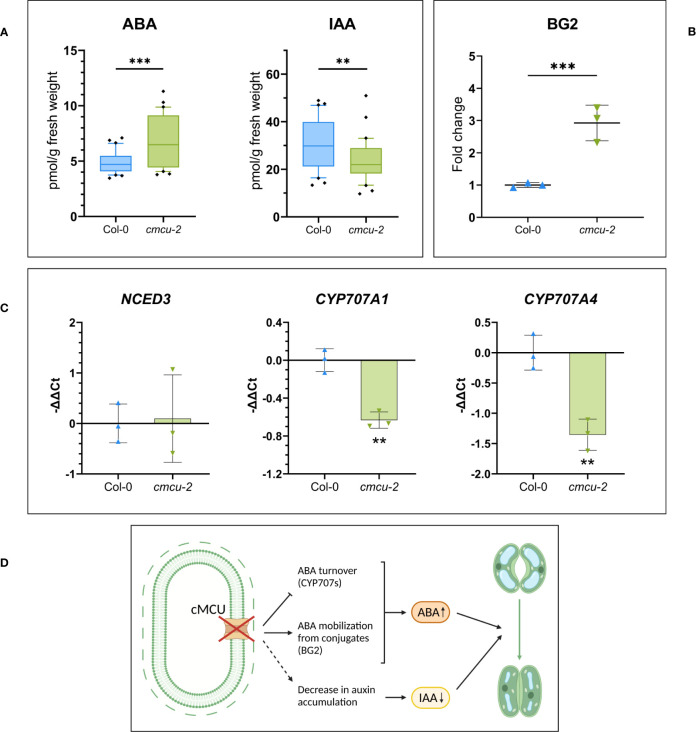
ABA and auxin level quantification in *cmcu-2* non-stressed plants. **(A)** Box plots of hormone quantification of ABA and IAA content in *cmcu-2* vs. Col-0 WT leaves (n=30), showing an increased content in ABA and a decreased content in IAA in non-stressed *cmcu-2* samples. **(B)** BG2 protein level increase in proteomics analysis, represented as fold-change in *cmcu-2* compared to Col-0 WT (mean ± SD, n=3), which may impact ABA mobilization from inactive conjugates in non-stressed *cmcu-2* samples. **(C)** Transcript level in RT-qPCR of NCED3 gene, rate-limiting in ABA *de novo* biosynthesis, and CYP707A1 and CYP707A4, involved in ABA degradation. Expression levels are reported as -ΔΔCt for Col-0 WT and *cmcu-2* (mean ± SD, n=3), and are normalized on average expression of the gene in Col-0 WT. **(D)** Summary of alterations of ABA and auxin accumulation emerging in *cmcu* plants and consequent altered regulation of stomatal closure. ** p<0.05; +*** p<0.01.

Given the marked increase in BG2 protein level in the *cmcu-2* plants, we performed a hormone quantification in control conditions (n=30 measurements from leaves of 3 different plants for each genotype, see **Supplementary Figure 2A**) in watered, unstimulated plants. From this analysis, a difference in auxin and ABA content emerged (**Figure 3A**), with the latter being consistent with the difference in protein content observed for BG2 (**Figure 3B**), suggesting a possible mechanism for the approximately 50% increase of ABA accumulation. To gain further insight into the possible mechanism of ABA increase in the mutants, we performed a quantitative real-time PCR (RT-qPCR) analysis for key, rate limiting proteins involved in ABA homeostasis (**Figure 3C**). 9-cisepoxycarotenoid dioxygenase (NCED) catalyses in the plastids the oxidative cleavage of 9-cis-violaxanthin and/or 9- cis-neoxanthin to produce xanthoxin, the rate rate-limiting step in ABA biosynthesis (Seiler et al., 2011). AtNCED3 is thought to play a major role in ABA biosynthesis in response to stress, while the transcript levels of cytochrome P450 CYP707As have been shown to be induced by dehydration (Kushiro et al., 2004). AtNCED3, AtCYP707A1 and AtCYP707A4 (abscisic acid 8′-hydroxylase 1 and 4) were all shown to be induced by osmotic stress (Chen et al., 2020) and involved in *de-novo* biosynthesis and degradation, respectively. In our case, the CYP707A1 and CYP707A4 transcripts were significantly downregulated in *cmcu-2* leaves compared to the WT, indicating that a lower ABA turnover rate may also contribute to higher hormone accumulation in *cmcu-2* plants. No significant differences were instead observed for NCED3 (**Figure 3C**). A graphical summary of the processes involved in altered ABA accumulation in the *cmcu-2* KO line is represented in **Figure 3D**.

Interestingly, in addition to ABA level, there was a significant, more than 50% increase also in the level of phaseic acid (PA) (**Supplementary Figure 2B**), a terpenoid catabolite of ABA whose level generally correlates with changes in ABA level [e.g (Kanno et al., 2010)]. We also measured the levels of auxin (indole-3-acetic acid (IAA)) in the *cmcu-2* plants compared to WT and found a significant decrease by approximately 25% of both IAA and oxindole-3-acetic acid (oxIAA), a degradation product of IAA (**Figure 3A**; **Supplementary Figure 2B**). These results are in agreement with the role of auxin in the guard cells, namely that of antagonizing ABA-induced stomatal closure [see e.g (Tanaka et al., 2006)]. Differences were not detected at the level of jasmonates (**Supplementary Figure 2B**) and of active forms of cytokinins, that can promote the stomatal opening, while a small but significant increase in the inactive N-and O-glucoside form of cytokinin was observed (**Supplementary Figure 2C**).

Since altered hormone levels might affect ion homeostasis [e.g (D'Oria et al., 2022; Keshishian et al., 2022)] and since cMCU-regulated Ca^2+^ signaling might alter ion transport, we performed a global ionome analysis by inductively coupled plasma optical emission spectrometry (ICP-OES). Total ion content was determined instead of performing ionomics on isolated chloroplasts, as ions would quickly move across the chloroplast envelope down their electrochemical gradient during isolation. Mineral nutrient, trace elements and the inorganic component of the plants constitutes the so-called “ionome”, which is an important determinant of the plant physiological state. Thus, we assayed Col-0 and *cmcu-2* plants for macronutrients (Ca, K, P, and S) and micronutrients (B, Cu) and other metal ions (Ba, Co, Fe, Mg, Mn, Zn) in order to observe possible differences in the total element content of leaves, compared to the WT. The only significant difference we observed was a higher sodium content in the plants lacking cMCU (**Supplementary Figure 3**). However, the Na^+^/K^+^ ratio, which could be important for salt tolerance [e.g (Chen et al., 2007)], was 0.0082 ± 9,9E-04 (mean ± SEM, n=3) in *cmcu-2* watered plants *versus* 0.00679 ± 5,6E-04 (mean ± SEM, n=3) in the Col-0, yielding a non-significant difference.

### 3.3 Photosynthetic activity in plants lacking cMCU

We have previously shown that *Arabidopsis* plants lacking cMCU are able to maintain their photosynthetic activity during long-term drought stress, and that short-term cMCU signaling is linked to photosynthetic activity and ROS release in two independent *cmcu* mutants, compared to their respective WT plants (on Col-0 and Col-4 backgrounds). In fact, a difference in the activation of MAPK3/6 upon mannitol addition to leaves, mimicking drought stress, was observed in *cmcu* plants compared to WT, but this occurred only when plants were illuminated. Moreover, treatment with N-acetyl cysteine, a cell permeable agent able to boost anti-oxidant activity, abolished the difference in *cmcu* plants. In addition, a differential regulation of MAPK3/6 downstream transcription factors occurred in *cmcu*, but the difference was abolished when photosynthesis was blocked by treatment with DCMU (Teardo et al., 2019). Taking into account these observations, we investigated more in depth the impact that chloroplast Ca^2+^ dynamics has on photosynthesis. In particular, we focused on the response to high light intensity and photoinhibition in both *cmcu-1* and *cmcu-2* lines under watered conditions.

**Figure 4:**
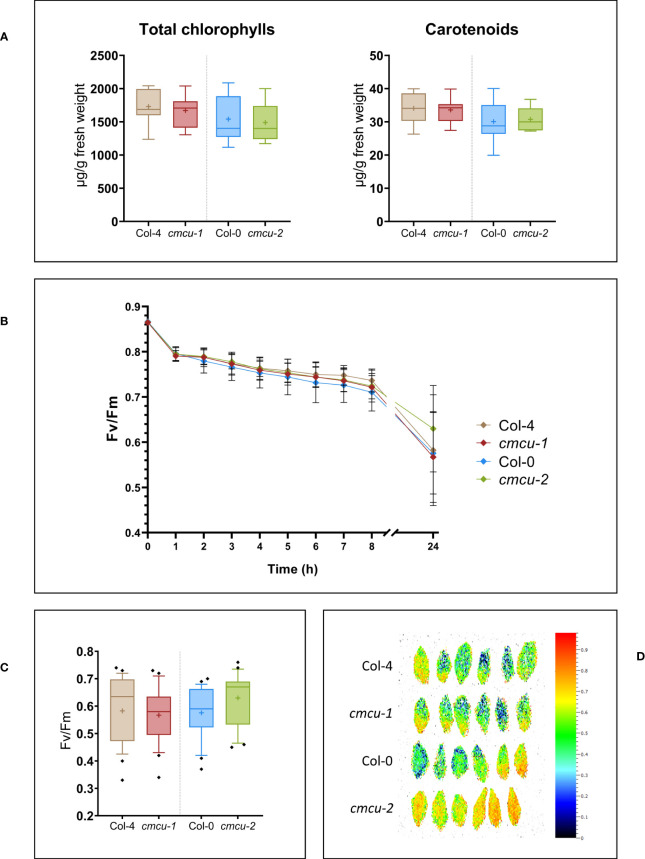
Characterization of pigment content and photosynthetic activity in *cmcu* plants. **(A)** Box plots of total chlorophylls and carotenoids quantification in *cmcu-1* and -2 leaf samples, compared to Col-4 and Col-0 WT (n=8, “+” shows the mean value). **(B)** Time-course of Fv/Fm measurement in fresh detached leaves, at time 0 and at different timepoints during exposure to high light at 1500 µmol photons m^-2^ s^-1^. Focusing on the last timepoint, **(C)** box plots of the Fv/Fm values (n=24) and **(D)** leaf images of Fv/Fm for 6 replicates representative for the total (n=24), after 24 hours of high light treatment at 1500 µmol photons m^-2^ s^-1^.

First, we assessed whether expression of *cMCU* affected resistance to short-term photoinhibition in WT and KO plants, which show comparable levels of chlorophyll and carotenoid content (**Figure 4A**). Illumination of leaves from the four different genotypes (Col-0 *versus cmcu-2* and Col-4 *versus cmcu-1*) with 1500 µmol photons m^-2^ s^-1^ light intensity caused a similar decrease in function of time of the Fv/Fm value, which is indicative of photosynthetic activity, **Figure 4B**). After 24 hours of illumination, no significant differences could be observed (**Figures 4C, D**; **Supplementary Figure 4**).

**Figure 5:**
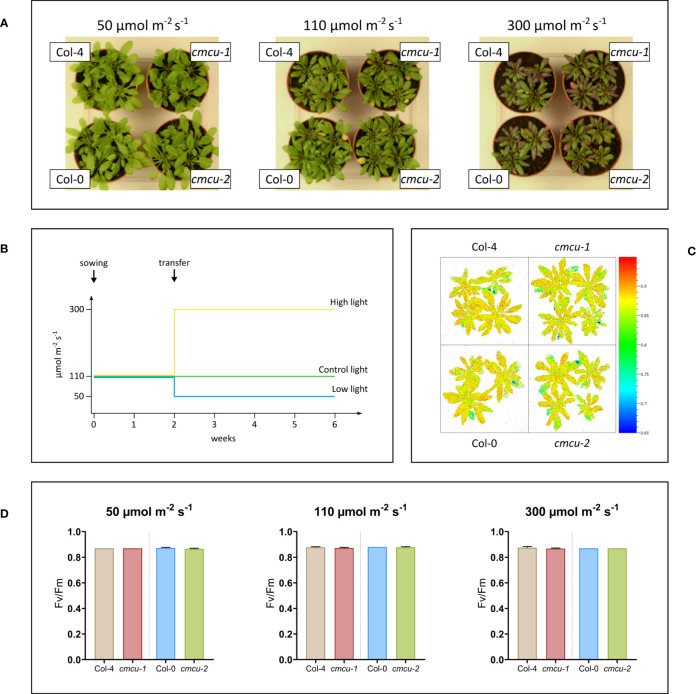
Growth phenotype investigation of *cmcu* plants under different light intensities. **(A)** WT and *cmcu* plants of 6 weeks of age, after 4 weeks of exposure to low (50 µmol photons m^-2^ s^-1^), control (110 µmol photons m^-2^ s^-1^) and high (300 µmol photons m^-2^ s^-1^) light intensity. **(B)** Diagram summarizing the light exposure of plants during growth. **(C)** Fv/Fm graphic representation for plants of 6 weeks of age, grown for 4 weeks at 300 µmol m^-2^ s^-1^ high light intensity. **(D)** Fv/Fm values (n=4, mean + SD) of Col-4, *cmcu-1*, Col-0 and *cmcu-2* for plants grown at different light intensities.

Next, we checked whether growth and photosynthetic activity of WT and KO plants at different light intensities were affected by the absence of the channel (**Figure 5A**). Plants were grown for 4 weeks at low (50 µmol photons m^-2^ s^-1^), normal (110 µmol photons m^-2^ s^-1^) and high light (300 µmol photons m^-2^ s^-1^) intensities starting from 2 weeks of age (**Figure 5B**), and rosette size as well as photosynthetic activity expressed as Fv/Fm were investigated (**Figure 5C**). As observable in **Figure 5**, no differences could be revealed.

### 3.4 Proteomic characterization of Col-0 and Col-4 WT ecotypes in watered condition

**Figure 6:**
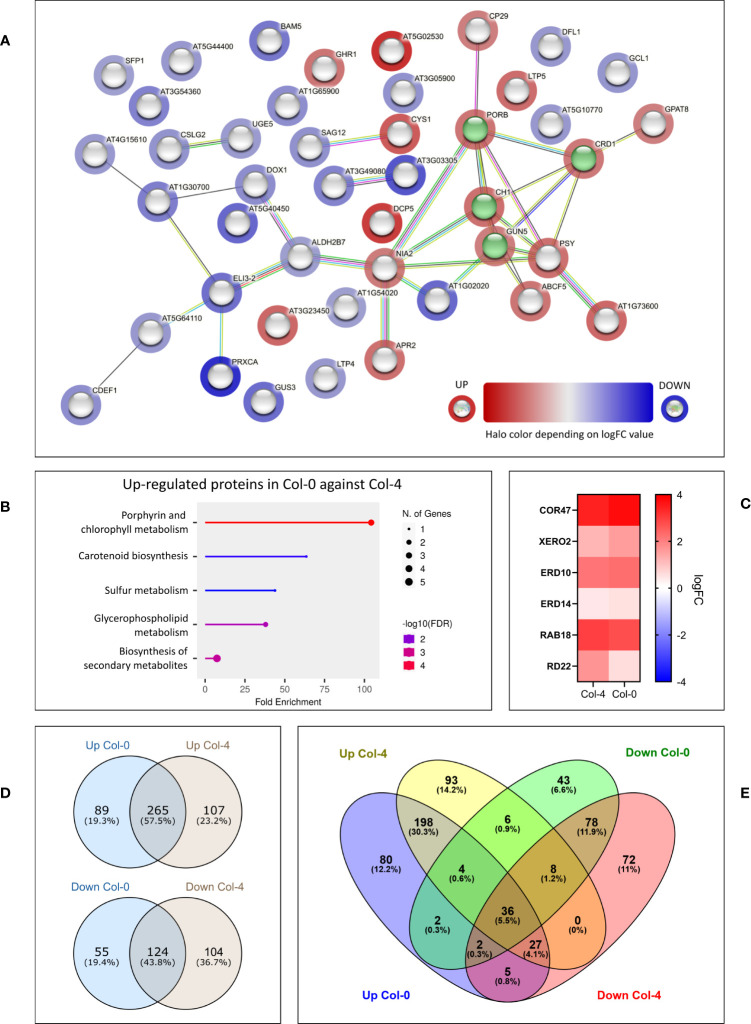
Differences emerging from proteomics between the Col-0 and the Col-4 ecotypes, both in non-stressed and in drought conditions. **(A)** STRING network of DEPs in Col-4 vs. Col-0 in non-stressed conditions. The logFC value for each protein is represented through the halo color. Proteins annotated by STRING as belonging to “chlorophyll biosynthetic process” class are highlighted in green. **(B)** ShinyGO chart representation of top enriched GO categories among up-regulated DEPs in non-stressed Col-0 vs. Col-4, showing fold enrichment, protein number and FDR values for each class. **(C)** Heatmap representing dehydrin upregulation pattern in Col-4 and Col-0 WT drought samples, compared to non-stressed ones. For each protein, the logFC values are shown in color scale. **(D, E)** Venn diagrams illustrating the overlap and differences in enriched GO classes found among either up- or down-regulated DEPs in Col-0 and Col-4, regarding drought vs. watered comparison. Enrichment analysis in the GO Biological Process ontology was performed with Panther and enriched GO classes were compared across Col-0 and Col-4 ecotypes. When comparing the list of proteins who were significantly upregulated in Col-4 drought vs. watered sample with the list of the proteins that were significantly downregulated in the Col-0 drought vs. watered samples and viceversa, we found 29 proteins that changed in the opposite direction (not shown).

Given the lack of constitutive difference in photosynthetic performance of plants either expressing or lacking cMCU, while significant differences are present in hormone levels, we performed another set of proteomic analysis. We studied the whole proteome from leaf samples of both *cmcu-1* and *cmcu-2*, compared with their corresponding WT ecotypes Col-4 and Col-0, using samples obtained both in control and drought stress conditions. In order to analyze data in a more stringent way, proteins were considered “hits” for a specific comparison if the fold change was over 2 (logFC > 1 or logFC < -1) and were considered “candidates” when the proteins had a fold change over 1.5 (logFC > 0.58 or logFC < -0.58). To begin with, we noticed that strong differences emerged between the Col-0 and Col-4 ecotypes, a topic not addressed so far to our knowledge (Mergner et al., 2020). Therefore, we first compared Col-0 and Col-4 proteome in watered conditions. We found a group of DEPs, represented in an interaction network using STRING in **Figure 6A**. The degree of up- (red) or downregulation (blue) in Col-4 *versus* Col-0 is represented for each DEP, while a green color marks proteins annotated by STRING as belonging to the GO class for chlorophyll biosynthetic process (GO:0015995). An enrichment in proteins involved in chlorophyll/pigment biosynthesis was detected among the upregulated proteins in Col-0 compared to Col-4 using the Panther tool (**Table 2**). The same result was also found using the ShinyGO tool, the results of which are presented in **Figure 6B**.

**Table 2:** Enriched GO Biological Process classes regarding chlorophyll/pigment biosynthesis found among DEPs in Col-0 vs Col-4 comparison.

GO-term	Biological process	Protein n. (in 17)	Fold enrichment	FDR
**GO:0015995**	chlorophyll biosynthetic process	4	> 100	2.09E-04
**GO:0006779**	porphyrin-containing compound biosynthetic process	4	81.70	3.52E-04
**GO:0033014**	tetrapyrrole biosynthetic process	4	71.71	3.49E-04
**GO:0015994**	chlorophyll metabolic process	4	50.82	1.10E-03
**GO:0046148**	pigment biosynthetic process	5	49.80	2.70E-04
**GO:0042440**	pigment metabolic process	5	34.04	4.27E-04
**GO:0006778**	porphyrin-containing compound metabolic process	4	29.20	8.09E-03
**GO:0033013**	tetrapyrrole metabolic process	4	24.36	1.43E-02

Reported results were obtained through Panther enrichment analysis.

### 3.5 Comparison of drought-stressed plant proteome reveals differential expression of proteins belonging to common pathways

Next, we compared the drought response of Col-0 and Col-4 ecotypes and of their respective mutants. First, we used Panther to detect DEPs in the Col-0 proteome in drought-stressed *versus* control condition, and likewise for Col-4. In both Col-0 and Col-4 plants there was a strong, up to 13-fold upregulation of known drought stress markers and dehydrins, and a similar pattern of upregulation for these markers was observed in both plant lines (**Figure 6C**), indicating that water deprivation indeed induced drought stress.

Also in this case, many differences among the enriched categories in Col-0 and in Col-4 were detected. Following the Panther analysis, which highlighted the enriched GO Biological Process classes in Col-0 and Col-4 in drought response, Venn diagrams were used to represent the common and distinct enriched categories in the two ecotypes (**Figures 6D, E**), revealing substantial differences in response to water deprivation in the two ecotypes. A complete list of the GO Biological Process classes found to be enriched in drought response in Col-0 and Col-4 can be found in **Supplementary Table 3**. Interestingly, abscisic acid biosynthetic process, several metabolic processes including parts of nucleotide and amino acid metabolism, ROS-related processes, translation and ribosome biogenesis, RNA processing and photoprotection processes had different enrichment patterns between the two ecotypes.

**Figure 7:**
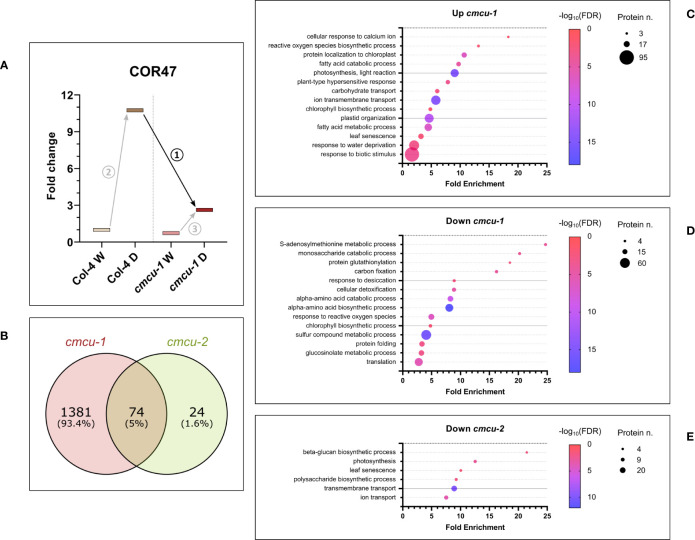
Proteomic analysis regarding the comparison of *cmcu* samples (n=3 for each line/condition) to their corresponding WT in drought response **(A)** Diagram illustrating the comparisons across samples that were taken into account for the analyses (see text for details). **(B)** Venn diagrams illustrating the overlap and differences in DEPs found in *cmcu-1* and *cmcu-2* in drought vs. watered comparison. Bubble plots showing representative enriched GO classes among up- **(C)** and down-regulated proteins **(D)** in *cmcu-1*, and among down-regulated proteins in *cmcu-2* **(E)**. Enriched GO classes in Biological Process ontology were obtained by Panther analysis, and their corresponding fold enrichment, protein number and FDR values are reported in the bubble plots. Please note that only few proteins belonging to the same GO categories that are regulated in an opposite way in the two *cmcu* lines coincide (see **Supplementary Tables 8, 10**).

Given the different response to drought of the two ecotypes, we considered to perform comparative analysis of the global proteome taking into account the drought response of the *cmcu-1* and *cmcu-2* lines, compared to their corresponding WT ecotypes. Thus, we focused on the comparisons between *cmcu*-2 and Col-0 drought samples, and between *cmcu*-1 and Col-4 drought samples. Also in this case, dehydration markers were upregulated to a similar extent across *cmcu*-1 and *cmcu*-2, showing that similarly to WT, *cmcu* lines were also subjected to drought stress (**Supplementary Table 4**). For this analysis, as illustrated as an example for the dehydrin COR47 (**Figure 7A**), on one hand we assessed the set of proteins that were significantly up- or down-regulated between drought samples of WT and *cmcu* plants (numbered as comparison 1), in order to highlight the proteins that are differentially expressed in drought when cMCU is lacking. On the other hand, we compared the set of proteins that were up- or down-regulated as a consequence of drought stress in WT (comparison 2) and in *cmcu* plants (comparison 3), in order to identify proteins that are specifically modulated in *cmcu* lines but not in the WT lines (*cmcu*-specifically altered proteins).

Regarding the first type of analysis (comparison 1), in which differential expressed proteins were identified by comparing each mutant with the respective WT in drought conditions, we found 1455 significantly up- or downregulated proteins (logFC ≥0.58 or ≤-0.58) in *cmcu-1* (**Supplementary Table 5**), while 98 DEPs were detected in *cmcu-2* (**Supplementary Table 6**). The DEPs in *cmcu-1* were compared to those found in *cmcu*-2 (**Figure 7B**). Among the consistently downregulated chloroplastic proteins upon drought stress, ferritin-1, a key iron storage protein, was present in both *cmcu* lines. We also noticed that among the proteins related to Ca^2+^ handling, the plastidial Ca^2+^ sensing receptor CAS (Li et al., 2022) was consistently less downregulated in both *cmcu*-1 and *cmcu-2* lines upon drought stress compared to their respective WT lines (1.1 and 1.67 fold changes for *cmcu-*2 vs Col-0 and *cmcu-1* vs Col-4 in drought conditions, respectively). Up- and downregulated proteins in *cmcu-1* and *cmcu-2* were analyzed to find enriched GO classes in the Biological Process Ontology. Full enrichment results obtained using Panther are shown in **Supplementary Table 7**: many enriched GO classes were found in *cmcu-1* DEPs, while no enrichment was detected by Panther among *cmcu-2* up-regulated proteins, likely because of the reduced number of DEPs we could detect. Representative GO classes were selected among the enriched ones in the Biological Process ontology, and enrichment results for up- and down-regulated proteins in *cmcu-1* and for down-regulated proteins in *cmcu-2* in drought were represented in bubble plots, shown respectively in **Figures 7C–E**. Moreover, proteins included in the most interesting GO classes for *cmcu-1*, according to Panther, are shown in **Supplementary Table 8** (up-regulated in *cmcu-1*) and **Supplementary Table 9** (down-regulated in *cmcu-1*), while those included in the most interesting GO classes for *cmcu-2* downregulated proteins are shown in **Supplementary Table 10**.

**Figure 8:**
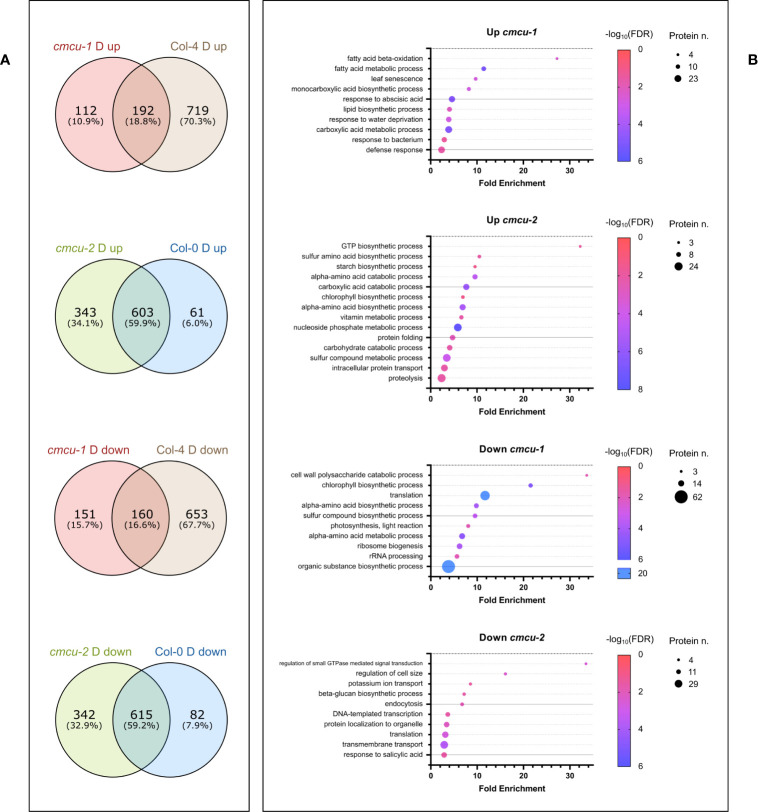
Further analyses of proteomics data for *cmcu* samples in drought response. **(A)** Venn diagrams illustrating the overlap and differences in DEPs found either in *cmcu-1* and Col-4, or in *cmcu-2* and Col-0 in drought vs. watered comparisons. Proteins exclusively found among *cmcu-1* (or *cmcu-2*) up- or down-regulated proteins in drought vs. watered comparison, and not in the corresponding WT in drought vs. watered comparison, were analyzed for enrichment in the GO Biological Process ontology with Panther. Representative enriched GO classes were represented with bubble plots **(B)** showing their corresponding fold enrichment, protein number and FDR values in either up- or down- regulated proteins in *cmcu-1* or *cmcu-2*.

The above analyses highlighted the differential expression of proteins that are up- or down-regulated upon drought stress in the *cmcu* lines with respect to their corresponding WT lines. In order to better understand which drought response-related proteins and pathways are specifically up- or down-regulated in the KO lines *versus* WT, we compared the set of DEPs found between drought-stressed and watered samples of Col-0 (comparison 2) with those identified between drought-stressed and watered samples of *cmcu-2* (comparison 3). The same analysis was performed for DEPs found in Col-4 in drought response compared with those present in *cmcu-1*. The Venn diagrams shown in **Figure 8A** reveal that 112 proteins are only up-regulated upon drought in the *cmcu-1* plants, and that 151 proteins are specifically down-regulated in this KO line, while in *cmcu-2* plants 343 proteins are specifically up-regulated and 342 are instead down-regulated. We assessed by Panther analysis the enriched GO Biological Process classes representing these *cmcu-1* and *cmcu-2*-specifically altered proteins under drought. Starting from full enrichment results reported in **Supplementary Table 11**, we selected representative GO enriched classes for either *cmcu-1* or *cmcu-2* specifically altered proteins and reported them in **Figure 8B**. Specific upregulated DEPs were linked to many biological processes, such as lipid metabolism, response to ABA and to water deprivation, response to biotic stress in *cmcu-1*, and to chlorophyll biosynthesis and many other metabolic pathways in *cmcu-2*. Among *cmcu-2*-specific DEPs, for example, LIN2, the chloroplastic coproporphyrinogen-III oxidase 1 (also known as CPX1), a key enzyme in the tetrapyrrole biosynthetic pathway for chlorophyll and heme, was significantly upregulated (logFC>0.7) in *cmcu-2* plants in contrast to WT upon drought stress. The same is true in *cmcu-2* for the 3’-phosphoadenosine 5’-phosphate (PAP) phosphatase SAL1 (Estavillo et al., 2011) that is importantly involved in drought stress response (Estavillo et al., 2011) and retrograde signaling (Pornsiriwong et al., 2017; Phua et al., 2018).

Moreover, an enrichment in chloroplast-localized proteins was found for these *cmcu*-specifically altered proteins by Panther analysis in the GO Cellular Component database. In particular, 65 out of 151 downregulated proteins in *cmcu-1*, 146 out of 343 upregulated proteins in *cmcu-2*, and 122 out of 342 downregulated proteins in *cmcu-2*, were annotated as chloroplast (GO:0009507) localized (**Table 3**). Additionally, since an enrichment for transcription- and translation-related processes was found in Panther Biological Process analysis both for *cmcu*-1 and *cmcu*-2 specifically downregulated proteins, we looked at the Molecular Function GO enrichment and found that downregulated proteins in *cmcu*-1 and -2 are also enriched for RNA/mRNA binding functions (**Table 4**).

Table 3Selection of enriched chloroplast-related terms in GO Cellular component among *cmcu-1* and *cmcu-2* exclusively up- and down-regulated proteins.

**Table:** 

cmcu-2 up				
GO-term	Cellular component	Protein n. (in 343)	Fold Enrichment	FDR
**(GO:0009507)**	**chloroplast**	146	2.48	3.54E-26
**(GO:0009526)**	plastid envelope	27	2.7	1.26E-04
**(GO:0009532)**	plastid stroma	62	7.1	1.59E-30
**(GO:0009536)**	plastid	166	2.6	7.09E-34
**(GO:0009570)**	chloroplast stroma	62	7.25	6.40E-31
**(GO:0009579)**	thylakoid	16	2.52	1.38E-02
**(GO:0009941)**	chloroplast envelope	24	3.39	1.71E-05
**(GO:0010319)**	stromule	6	13.23	2.70E-04

**Table:** 

cmcu-1 up *enrichment not detected* cmcu-2 down				
GO-term	Cellular component	Protein n. (in 342)	Fold Enrichment	FDR
(GO:0009507)	**chloroplast**	122	2.01	8.01E-13
(GO:0009534)	chloroplast thylakoid	13	2.44	3.75E-02
(GO:0009536)	plastid	134	2.04	5.11E-15
(GO:0009706)	chloroplast inner membrane	7	9.29	3.95E-04
(GO:0009707)	chloroplast outer membrane	5	7.34	1.16E-02
(GO:0009941)	chloroplast envelope	35	4.81	9.02E-12
(GO:0031969)	chloroplast membrane	12	7.6	4.61E-06
(GO:0042644)	chloroplast nucleoid	7	11.95	1.00E-04

**Table:** 

cmcu-1 down				
GO-term	Cellular component	Protein n. (in 151)	Fold Enrichment	FDR
(GO:0009507)	**chloroplast**	65	2.44	1.70E-11
(GO:0009521)	photosystem	4	11.91	9.85E-03
(GO:0009526)	plastid envelope	19	4.2	6.30E-06
(GO:0009532)	plastid stroma	30	7.61	7.12E-16
(GO:0009534)	chloroplast thylakoid	9	3.85	1.36E-02
(GO:0009535)	chloroplast thylakoid membrane	8	4.42	1.19E-02
(GO:0009536)	plastid	70	2.43	1.66E-12
(GO:0009570)	chloroplast stroma	30	7.77	4.40E-16
(GO:0009941)	chloroplast envelope	13	4.07	6.90E-04

Table 4Enriched GO Molecular Function terms regarding RNA and mRNA binding among *cmcu-1* and *cmcu-2* exclusively down-regulated proteins.

**Table:** 

cmcu-1 down				
GO-term	Molecular function	Protein n. (in 151)	Fold Enrichment	FDR
**(GO:0003723)**	RNA binding	48	5.64	4.30E-20
**(GO:0003729)**	mRNA binding	47	8.46	1.47E-26

**Table:** 

cmcu-2 down				
GO-term	Molecular function	Protein n. (in 342)	Fold Enrichment	FDR
**(GO:0003723)**	RNA binding	51	2.63	5.10E-07
**(GO:0003729)**	mRNA binding	40	3.16	4.97E-07

### 3.6 Drought-stressed cmcu plants display a change in time-dependent gene expression

**Figure 9:**
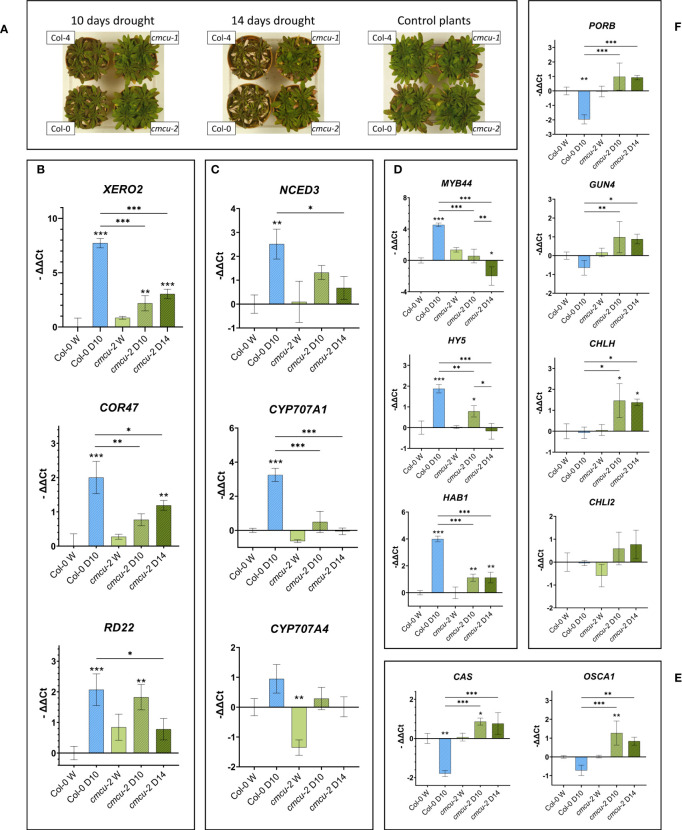
RT-qPCR expression results for genes of interest in *cmcu-2* and Col-0 watered and drought-stressed samples **(A)** WT and *cmcu* plants after 10 or 14 days of drought, together with non-stressed, control plants. **(B-F)** Transcript level in RT-qPCR of drought marker genes **(B)**, genes involved in ABA *de novo* biosynthesis and degradation **(C)**, other genes of interest involved in stress response (D and E) and genes encoding chlorophyll biosynthesis pathway proteins **(F)**. Expression levels are reported as -ΔΔCt (mean ± SD, n=3) for Col-0 WT and *cmcu-2* leaves in watered (marked as W) and drought stress conditions, lasting either 10 or 14 days (marked as D10 and D14). Asterisks on bars are used to highlight significant differences of the sample with respect to the control (Col-0 watered sample), while asterisks on horizontal lines highlight significant differences in other comparisons.

In our exploratory proteome analysis upon drought stress we found proteins belonging to photosynthesis and chlorophyll biosynthesis pathway, retrograde signaling, dehydrins or to other types of drought-responsive proteins. We decided to further investigate the time-dependent expression of some of the genes encoding for these proteins by RT-qPCR both in control and drought stressed samples. In this independent set of plants (**Figure 9A**), the samples were taken from the WT plants after 10 days of water deprivation and after both 10 and 14 days for the *cmcu* plants (WT plant samples were not suitable for analysis on day 14 of water deprivation). First, we assessed the expression of typical drought markers in the plants subjected to water deprivation. Graphs in **Figure 9** refer to Col-0 *versus cmcu-2*, but similar results were obtained also in Col-4 *versus cmcu-1* (**Supplementary Figure 5**). Interestingly, the mRNA levels of XERO2 and COR47 dehydrins increased more slowly in the *cmcu* than in WT plants (**Figure 9B**). The expression of another drought marker, RD22, mirrored the situation observed with dehydrins but showed a maximal expression at day 10 in the KO.

Next, we studied the expression of several genes whose protein products were found as DEPs in the proteome analysis. First, we explored the expression level of genes linked to ABA homeostasis (NCED3, CYP707A1 and CYP707A4), observing that i) the gene expression of the rate-limiting ABA *de-novo* biosynthesis enzyme NCED3 is not as upregulated in drought in *cmcu* plants as it is in the WT; and ii) the gene expression of CYP707A1 and CYP707A4, enzymes involved in ABA degradation, is not upregulated in drought in *cmcu* mutants while it is in the WT (**Figure 9C**; **Supplementary Figure 5**). These results are in agreement with the enhanced ABA level in *cmcu* plants and with data presented in **Figure 3C** for *cmcu-*2 under watered condition. Expression of the dehydration-activated MYB44 transcription factor, which is transcriptionally activated by ABA and confers drought tolerance through stomatal closure (Jung et al., 2008), was surprisingly lower in the KO plants compared to WT (**Figure 9D**). The same tendency was observed for the transcription factor ELONGATED HYPOCOTYL 5 (HY5), that connects light and stress signals (Kovács et al., 2019) and seems to interact with ABI5 ensuring ABA signaling integration (Bhagat et al., 2021) (**Figure 9D**). The protein phosphatase type 2C HAB1, one of the key negative regulators of ABA signaling (Saez et al., 2004), was also much less upregulated in the mutant plants compared to WT plants (**Figure 9D**).

We also assessed the expression level of two genes whose protein products are crucial for stress responses, the plastidial Ca^2+^ sensor CAS (Li et al., 2022) and OSCA1, an osmosensing calcium-permeable channel that controls stomatal closure (Yuan et al., 2014; Thor et al., 2020) (**Figure 9E**), revealing in both cases an up-regulation of gene expression in *cmcu-2* samples, but not in WT, upon drought, highlighting an apparent de-repression in *cmcu* plants. Finally, we found significant differences in some of the chlorophyll biosynthesis genes such as PORB, GUN4, CHLH and CHLI2 (**Figure 9F**). GUN4 and CHLH (also known as GUN5) are additionally known to take part in retrograde signaling (de Souza et al., 2017). Strikingly, GUN4, CHLH and CHLI2 were not upregulated upon water deprivation in the WT plants, while a significant increase occurred both in the *cmcu-2* line (**Figure 9F**) and in *cmcu*-1 line (**Supplementary Figure 5**).

### 3.7 Bioinformatic analysis of putative interactor network of cMCU

**Figure 10:**
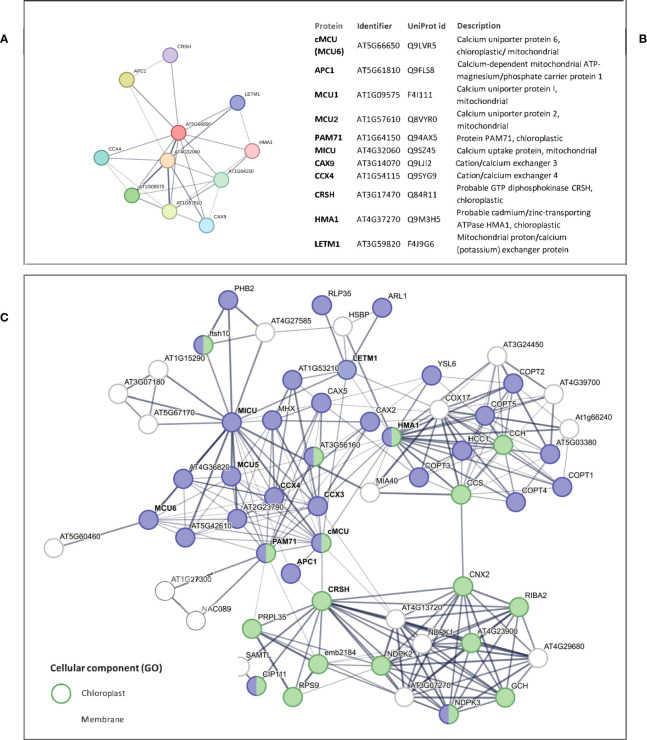
Putative interactor network of cMCU according to interaction databases. **(A)** STRING network including *Arabidopsis* cMCU and its 10 best putative interactors, with corresponding protein identifier and description reported in **(B)**. **(C)** STRING network including *Arabidopsis* cMCU, its 10 best putative direct interactors (names in bold) and 50 additional indirect interactors. STRING annotations for proteins belonging to “chloroplast” and “membrane” GO Cellular Component classes are highlighted in green and blue, respectively.

Last, we explored the possibility that the putative interaction partners of cMCU identified by STRING network analysis may contribute to the altered signaling events we observed in the *cmcu* plants. The predicted interaction network for *Arabidopsis* cMCU is shown in **Figures 10A, B**, where, among the 10 predicted best direct interactors, we could find HMA1, a chloroplastic probable Cd^2+^/Zn^2+^-transporting ATPase, CRSH, a Ca^2+^-activated plastidial RelA/spot-like protein that possesses Ca^2+^-dependent ppGpp (guanosine 3’-diphosphate 5’-diphosphate) synthetase activity and has been linked to drought stress (Ono et al., 2021), the putative Ca^2+^/Mn^2+^ transporter BICAT1 (also named PAM71) (Schneider et al., 2016; Frank et al., 2019), the endomembrane localized cation/calcium exchanger CCX3, other MCU isoforms, and a putative peroxisomal Ca^2+^-dependent solute carrier protein and cation/calcium exchanger CCX4 and LETM1, a putative K^+^/H^+^ exchanger involved in Ca^2+^ signaling modulation (Austin et al., 2017). Among the putative partners, we identified in our proteome LETM1 and HMA1, which however did not show significant changes in expression level among WT and mutants and among watered and drought samples (not shown). However, by extending the list of predicted interactors to indirect ones (**Figure 10C**), we could interestingly find nucleoside diphosphate kinases 1, 2 and 3 (NDPK1, -2, -3). While NDPK3 shows chloroplastic/mitochondrial localization, NDPK2 is plastidial only (Bölter et al., 2007) and has been proposed to activate MAPK3 and MAPK6 (Moon et al., 2003).

## 4 Discussion

In the present study we followed up on our previous findings showing an enhanced drought resistance in *Arabidopsis* plants lacking cMCU compared to WT plants (Teardo et al., 2019). The purpose of this paper was to understand whether i) differences in protein expression, hormone levels and ionome under basal, non-stressed conditions could be detected in the mutant plants compared to WT lines; ii) the mutant plants responded in a different way regarding regulation of protein expression upon drought stress compared to the WT plants. The results of these analyses are discussed taking into account information available in the literature regarding drought-stress response in *Arabidopsis* and crop species.

As described above, significant differences in protein expression were found between *cmcu* and WT plants at constitutive level in plants grown in the same growth chamber under the same conditions. One of the most striking differences we observed was the upregulation of BG2 (At3g57260). ABA can be inactivated by conjugation with ABA-Glc ester (ABA-GE), one of the predominant ABA conjugates representing the releasable form of ABA, is hydrolyzed in response to dehydration by β-glucosidases (AtBG1/AtBG2), leading to an increase in the active ABA pool (Lee et al., 2006). It has been reported that an increase in BG1/2 expression and/or activity is linked to enhanced ABA levels upon dehydration stress through a mechanism called polymerization-mediated activation (Xu et al., 2012). Both *bg1* and *bg2* plants showed and increased water loss and were proposed to contribute independently to the dehydration stress response (Xu et al., 2012). In *Arabidopsis* BG1 and BG2 have been located to the ER and vacuoles, respectively (Xu et al., 2012), although in a more recent paper also AtBG2 was found to be located in the ER (Zavaliev et al., 2013), raising the possibility of a control of its expression level by chloroplast Ca^2+^ dynamics, given that a communication among ER and chloroplasts is of relevance in various contexts (see e.g. Costa et al., 2018; Liu and Li, 2019). Interestingly, a recent study identified a chloroplastic β-glucosidase isoenzyme in rice, Os3Bglu6, that functions in ABA recycling (Wang et al., 2020). The authors showed that the lack of this enzyme in rice resulted in lower ABA content and photosynthesis rate in leaves, in impaired stomatal movement and in higher drought-sensitivity. The mechanism by which photosynthesis was lowered, however, has not been clarified. Similar to *Arabidopsis* BG2, overexpression of Os3Bglu6 decreased stomatal aperture under both normal and drought conditions and increased the drought tolerance, ABA concentrations and expressions of ABA- and drought-responsive genes. Furthermore, transcriptomics revealed that disruption of Os3Bglu6 resulted in chloroplastic oxidative stress. Among the 32 predicted genes encoding β-glucosidase enzymes in *Arabidopsis*, only At3g07320 shows a predicted localization to chloroplasts (see Aramemnon database, http://aramemnon.uni-koeln.de/) (Schwacke et al., 2003). However, no experimental evidence on the function and localization of this protein is available, and this protein was not detected in our proteomic analysis. In summary, our findings of an upregulated BG2 protein level (**Figure 3B**), as well as of a downregulation of the transcript of ABA-degradation enzymes (**Figure 3C**) match well the observed constitutively higher ABA level (see **Figure 3A**) and partially closed stomata (Teardo et al., 2019) in *cmcu* plants. Interestingly, in a previous study that was investigating the genes of unknown function with abiotic stress responses by high-throughput phenotype screening, one SALK t-DNA insertion line for At5g66650 (defined as gene with unknown function) showed an enhanced tolerance to ABA, however the mechanism has not been investigated (Luhua et al., 2013).

In addition to BG2, the highly upregulated proteins include a so-far poorly characterized transducing/WD-40 repeat family protein, Aspartyl protease AED1 (Apoplastic EDS1-dependent protein 1), as well as calreticulin-3 (CRT3) and plant cadmium resistance 1 protein (PC1). WD40 proteins are involved in various stress responses also through positive regulation of ABA response and by stabilizing accumulation of the transcription factor ABA Insensitive 5 (ABI5) (Xu et al., 2019), while AED1 has been proposed to be part of a homeostatic feedback mechanism regulating systemic immunity (Breitenbach et al., 2014), a process linked to plastidial Ca^2+^ dynamics (Nomura et al., 2012). CRT3, an ER-located Ca^2+^-binding chaperone, is encoded by a gene that is co-expressed with genes involved in plant defense, was proposed to be responsible for retaining misfolded glycoproteins in the ER under various stress conditions (Jin et al., 2009) and seems to exert a similar function in rice (Thelin et al., 2011). *Crt3* plants are more sensitive to low Ca^2+^ and salt stress compared to WT plants (Zhigang et al., 2008) and expression of the C-domain of calreticulin, which shows low-affinity, high capacity Ca^2+^ binding in the ER, conferred better resistance to drought (Tsou et al., 2012). Interestingly, CRT3 also appears to mediate folding of the elf18 responsive EF-Tu receptor (EFR) associated with Pathogen-Associated Molecular Patterns (PAMPs) (Thelin et al., 2011), which is intimately linked to chloroplast Ca^2+^ dynamics (Nomura et al., 2012; Littlejohn et al., 2021). Altogether, the upregulation of CRT3 in the *cmcu* mutant suggests that beside the plastid calcium sensor CAS, cMCU might also be involved in plant immunity, a hypothesis that remains to be tested.

Among the constitutively downregulated proteins, we found RH3, RH26 and RH50, three plastid-located DEAD-box RNA helicases (RHs), which are enzymes that can alter RNA structures and thereby modulate interaction with their target proteins, affect RNA metabolism and are linked to abiotic stress response (Nawaz and Kang, 2017). The chloroplast-localized RH3 was reported to be essential for chloroplast ribosome biogenesis, carbon fixation and the maintenance of ABA level in *Arabidopsis* under environmental stresses, such as dehydration (Lee et al., 2013). Moreover, this factor functions in the splicing of group II introns and possibly also contributes to the assembly of the 50S ribosomal particle both in *Arabidopsis* and *Zea mays* (Asakura et al., 2012). AtRH26 was one of the RH factors whose mutations caused a decreased virus accumulation in the Turnip mosaic virus (TuMV)-*Arabidopsis* pathosystem (Li et al., 2016), while AtRH50 was shown to be a plastidial rRNA maturation factor required for efficient translation of plastid proteins (Paieri et al., 2018). Interestingly, in this latter work the authors demonstrated that RH50 and GUN1, which is involved in retrograde signaling, are functionally related and that this function is associated with plastid gene expression through modulation of ribosome functioning. How Ca^2+^ affects the expression level of these RNA helicases is unknown, but, interestingly, potassium ion homeostasis alteration within the chloroplasts has recently been shown to affect maturation of the plastid ribosomal RNAs, likely by modulating binding of RNA-processing proteins (DeTar et al., 2021). The resulting disturbance of plastid gene expression was shown in the same study to trigger GUN1-mediated retrograde signaling and to delay chloroplast biogenesis. In addition, a mutant downregulated for all six MCU homologs displayed differentially expressed genes in comparison to WT plants related to ribosome biogenesis and translation (Zhang et al., 2022).

As mentioned, *cmcu* plants were shown to be more resistant to drought stress than WT plants, a result that we confirmed in the present study. Altered plastidial Ca^2+^ dynamics either in *cas* (Guo et al., 2016) or *cmcu* plants (Teardo et al., 2019) was shown to affect retrograde signaling, in particular MAPK3/6 signaling in the cytosol. Guo et al. proposed that a Ca^2+^ exit from the chloroplast may activate MAPK via modulation by 14-3-3 proteins that would act as Ca^2+^-dependent scaffolds for MAPK activation (Guo et al., 2016). In the case of *cmcu* plants, the Ca^2+^ uptake into the plastids was reduced. Among the putative partners of cMCU we can find the Ca^2+^-dependent plastid-located CRSH, a Ca^2+^-dependent enzyme that catalyzes the synthesis of the signaling nucleotides guanosine penta- and tetraphosphate (ppGpp), a class of molecules involved in stress response and acting as a potent regulator of chloroplast gene expression that reduce steady state transcript levels in the chloroplast (Sugliani et al., 2016). CRSH is an interactor of NDPK2 that was shown to activate MAPK3/6 (Moon et al., 2003). Thus, it is tempting to speculate that cMCU affects CRSH function and/or expression, which in turn activates (plastidial) NDPK2 that transmits the signal to (cytosolic) MAPK3/6. Future experiments are required however to prove this hypothetical chain of events.

Beside an impact of cMCU on the short-term osmotic stress response involving MAPK3/6, we observed that on the longer term, *cmcu* plants showed resistance to water deprivation and maintained high photosynthetic activity. Our proteomic analysis combined with targeted transcript measurement revealed a de-repression of players of chlorophyll biosynthesis, including those involved in retrograde signaling. Importantly, the changes in transcript levels for these proteins have been confirmed in both *cmcu* lines, independently of the genetic background, even though, as reported in the present study, there are significant differences in the drought stress response between Col-0 and Col-4 WT plants. This result is not surprising, as for example a proteomic study involving Ws and Col-0 (Leschevin et al., 2021) revealed differences in proteins related to photosynthesis, cell wall-related proteins, plant defense and stress, ROS scavenging and redox homeostasis, DNA/RNA binding, transcription, translation and protein folding, likely accounting for the differential plant development and responses to environmental changes in the two ecotypes. The observation that we identified CAS and proteins involved in chlorophyll biosynthesis as DEPs and DEGs in both *cmcu* lines, with Col-0 and Col-4 backgrounds, and that the lines showed a highly similar drought resistance, strongly indicates that these players might be strictly linked to drought resistance in both *cmcu* lines.

Regarding the chloroplast-located Ca^2+^-sensing receptor CAS, its steady upregulation was shown to enhance stomata closure and drought tolerance (Nomura et al., 2008; Weinl et al., 2008; Zhao et al., 2015). Our finding, i.e. an increase in the CAS transcript and protein levels in both *cmcu* lines compared to WT lines upon drought stress, is in good agreement with the observed drought tolerance of the plants lacking cMCU. CAS was shown to be involved in the generation of cytosolic Ca^2+^ signals upon extracellular Ca^2+^ increase, in both *Arabidopsis* (Nomura et al., 2008; Weinl et al., 2008) and rice (Zhao et al., 2015), but its overexpression alone was sufficient to promote stomatal closure even in the absence of external calcium. Interestingly, the C-terminal part of CAS can be phosphorylated by STN7/STN8 (Cutolo et al., 2019). It has been proposed that, as a consequence, Ca^2+^ sequestered by CAS could be released into the chloroplast stroma (Li et al., 2022). In our proteomic analysis we revealed a differential regulation of STN7/8 upon drought stress between *cmcu* and WT samples, suggesting a possible link between cMCU, STN7/8 and CAS function. However, future work is required to understand the interplay, if any, between CAS and cMCU. In the work of Guo and colleagues, it was shown that CAS-mediated activation of MAPK3/MAPK6 induced activation of ABI4, which in turn repressed LHCB expression (Guo et al., 2016). CAS level is maintained high in the *cmcu* lines upon drought stress and we found for both CP26 and CP29 internal antenna LCHB proteins of photosystem II an enhanced downregulation upon drought stress in the *cmcu-2* line compared to Col-0 (logFC values of -1.6 and -1.8 in Col-0 drought *versus* watered, while values of -2.3 and -2.5 in *cmcu-2*), suggesting that similarly to CAS, cMCU might also regulate LHCB repression through MAPK3/6 activation. OSCA1, a hyperosmolarity-gated Ca^2+^-permeable channel was found to be de-repressed beside CAS in our study in both *cmcu* lines upon water deprivation. Sorbitol-induced stomatal closure was much reduced in *osca1* plants (Yuan et al., 2014). Interestingly however, ABA-induced stomatal closure was unaffected in *osca1*, suggesting an action of OSCA1 upstream of ABA (Yuan et al., 2014) and also independently of ABA in the context of PAMPs (Thor et al., 2020). Overexpression of *Oryza sativa* OsOSCA1.4 in *Arabidopsis osca1* mutant was shown to complement osmotic Ca^2+^ signaling and stomatal movement in response to hyperosmolality (Zhai et al., 2020). In addition, soybean OSCA isoforms also rescued the drought-hypersensitive phenotype of *osca1* (Liu et al., 2022). Altogether, the de-repression of OSCA1 expression in our study in the *cmcu* lines might also in part account for the drought-tolerance of *cmcu* plants.

Regarding other players of retrograde signaling in addition to CAS, we found an increased, de-repressed transcript level in both *cmcu* lines upon drought stress for genes whose protein products are involved in chlorophyll synthesis, i.e. PorB, GUN4, CHLH (GUN5), CHLI2. A de-repressed chlorophyll biosynthesis pathway is consistent with the ability of *cmcu* plants to maintain high photosynthetic activity even after prolonged water deprivation (Teardo et al., 2019). We focused our attention in particular on these enzymes because they have been linked not only to chlorophyll synthesis but also retrograde signaling and ABA cross-talk during drought stress response (Du et al., 2012). Both the H and the I subunits of Mg-chelatase (CHLH and CHLHI, respectively) were shown to regulate ABA signaling in guard cells. ABA was shown to bind to CHLH at least *in vitro* and downregulation of CHLI conferred ABA insensitivity in stomatal response in *Arabidopsis* and in tobacco (Du et al., 2012). Interestingly, overexpression of CHLH in *Arabidopsis* guard cells resulted in drought tolerance by induction of stomatal pore closure (Tsuzuki et al., 2013). Although further experiments are required to prove a possible connection between cMCU and retrograde signaling through GUN4 and GUN5 and possibly SAL1 (see above), our result is consistent with the drought-tolerant phenotype of *cmcu* plants.

Altogether, our data suggests that the lack of cMCU triggers a profound re-programming in the expression of several genes/proteins that might explain the drought tolerance of these *Arabidopsis* KO plants. This result is in agreement with the well-known ability of plants to remodel the transcriptional network and physiological processes as long-term response to water deficiency, beside the osmotic stress signaling cascade, which induces short-term cellular responses to reduce water loss. Several studies addressed the role of MCU isoforms in other plants as well. For example, promoter analysis also uncovered the existence of two canonical cold-related *cis*-acting elements in *MCU* and interestingly in the promoter region of soybean *MCU* isoforms stress-related phytohormone-responsive elements were found (Li et al., 2022). MCU homolog was also identified in pollen tubes of tobacco, where its role remains unclear (Flores-Herrera et al., 2019), and in pear (Wang et al., 2018). To our knowledge, no studies have addressed so far the role of MCU homologs in response to water deprivation in plants other than *Arabidopsis*. AtMCU1/2/3 were reported to set mitochondrial calcium uptake in the roots and be linked to regulation of jasmonic acid-related signaling and thigmomorphogenesis (Ruberti et al., 2022). AtMCU2 function was also linked to pollen tube germination (Selles et al., 2018), but in none of these works an potential role of MCU homologs in drought response was studied. Chloroplastic cMCU has different orthologs according to TAIR (**Supplementary Table 12**). However, we could not find a correspondence between *Arabidopsis thaliana* and the available *Oryza sativa* predicted networks (**Supplementary Figure 6**). Thus, future work is required to reveal the relevance of chloroplast-located calcium channels and related signaling networks in crops.

## Data availability statement

The original contributions presented in the study are included in the article/**Supplementary Material**. Further inquiries can be directed to the corresponding author.

## Author contributions

IS, LN, EF FS, ON and FC designed experiments. FC, FS, MF, PS, JS performed experiments. IS, FC, FS, UCV analysed data. IS and FC wrote the manuscript. UCV, EF and AA edited the manuscript. IS, LN, EF, UCV acquired funding. All authors contributed to the article and approved the submitted version.

## Supplementary material

Supplementary Figure 1 RT-qPCR expression results for genes belonging to the MCU family as measured in *cmcu-1*, *cmcu-2* and Col-0 as well as Col-4 samples. Samples obtained from regularly watered, 5 weeks-old plant leaves have been used for transcript analysis. Expression levels are reported as - ΔCt (mean ± SD, n=3), normalized on ACT2 expression in each biological sample and reported relatively to the lowest Ct found. There are no statistically significant differences between the expression level of the shown MCUs in the 4 lines. The primers used for the different genes were those employed in the study of Ruberti et al. (2022), see also **Supplementary Table 1**.

Supplementary Figure 2 Additional results of hormone quantifications (n=30). (A) Col-0 and *cmcu-2* plants sampled for hormone quantifications. (B) Box plots of hormone abundance for molecules in the jasmonate (JA, cis-OPDA and Ja-Ile), ABA (ABA, PA) and auxin (IAA, oxIAA) families, together with total cytokinins abundance (n=30). **(C)** Box plots of hormone abundance for molecules in the cytokinin family.

Supplementary Figure 3 Ionomic analysis of Col-0 and *cmcu-2* non-stressed (watered, W, n=3) and drought-stressed (D, n=2) plants. Element concentration in plants rosettes was normalized on plant tissue dry weight, and reported as mean + SD.

Supplementary Figure 4 Leaf images of Fv/Fm for all replicates (n=24) shown in the photoinhibition experiment shown in , after 24 hours of high light treatment at 1500 photons µmol m^-2^ s^-1^. Leaf genotype is indicated in the table below images.

Supplementary Figure 5 RT-qPCR expression results for genes of interest in *cmcu-1* and Col-4 watered and drought-stressed samples. Transcript level of drought marker genes **(A)**, genes involved in ABA *de novo* biosynthesis and degradation **(B)**, other genes of interest involved in stress response **(C, D)** and genes encoding chlorophyll biosynthesis pathway proteins **(E)**. Expression levels are reported as -ΔΔCt (mean ± SD, n=3) for Col-4 WT and *cmcu-1* leaves in watered (marked as W) and drought stress conditions, lasting either 10 or 14 days (marked as D10 and D14). Asterisks on bars are used to highlight significant differences of the sample with respect to the control (Col-4 watered sample), while asterisks on horizontal lines highlight significant differences in other comparisons.

Supplementary Figure 6 Putative interactor network of MCU homolog in *Oryza sativa* OS10T0501500-01 according to STRING interaction database.
