# Supplementary figures and images for: Comparative analysis of wild-type and chloroplast MCU-deficient plants reveals multiple consequences of chloroplast calcium handling under drought stress
Source: Front Plant Sci. 2023 Aug 25;14:1228060. doi: 10.3389/fpls.2023.1228060 (PMC10485843; doi:10.3389/fpls.2023.1228060)

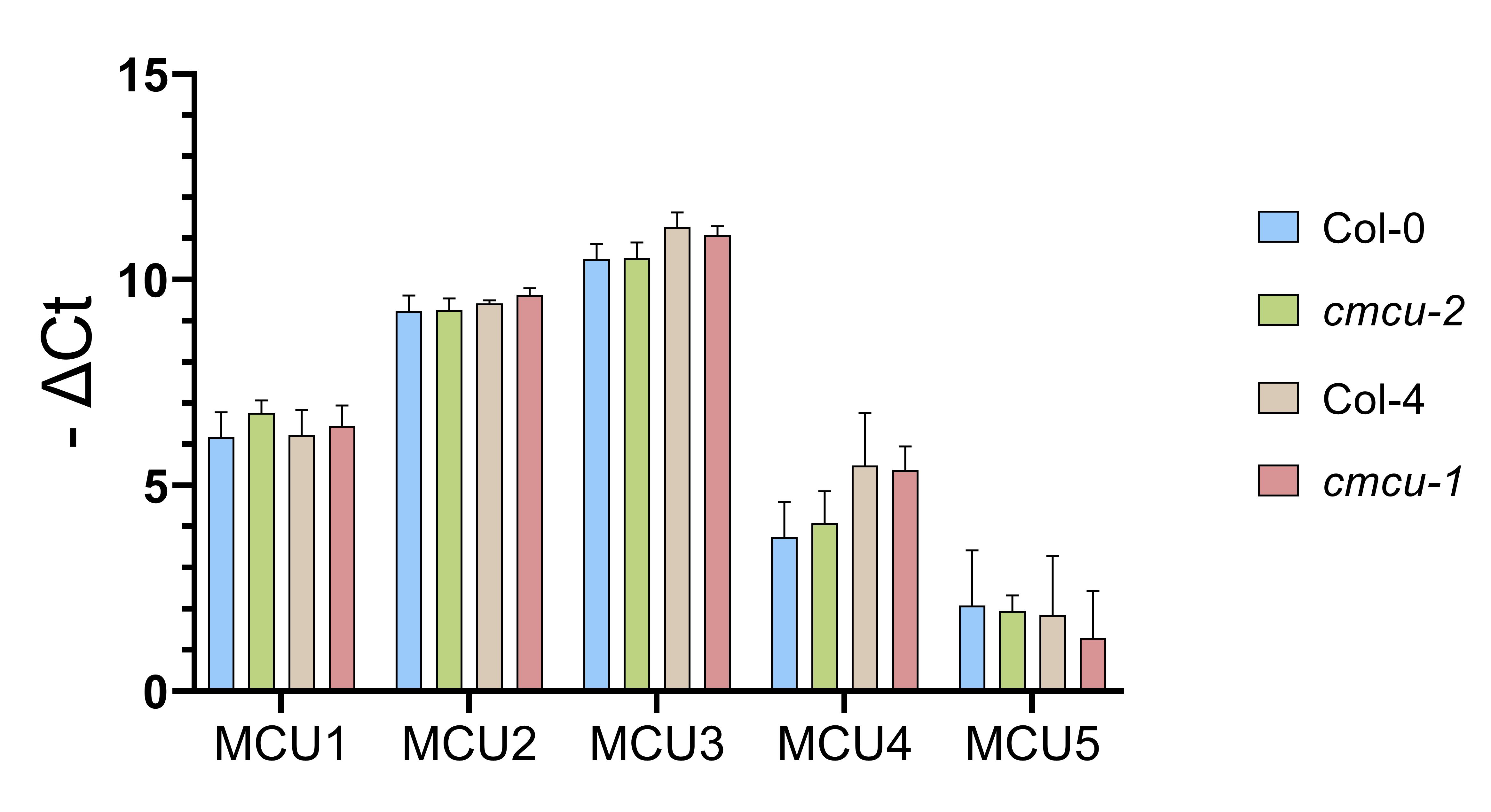

Supplement: Supplementary Figure 1 — RT-qPCR expression results for genes belonging to the MCU family as measured in cmcu-1, cmcu-2 and Col-0 as well as Col-4 samples. Samples obtained from regularly watered, 5 weeks-old plant leaves have been used for transcript analysis. Expression levels are reported as - ΔCt (mean ± SD, n=3), normalized on ACT2 expression in each biological sample and reported relatively to the lowest Ct found. There are no statistically significant differences between the expression level of the shown MCUs in the 4 lines. The primers used for the different genes were those employed in the study of Ruberti et al. (2022), see also Supplementary Table 1 . [file Image_1.jpeg]

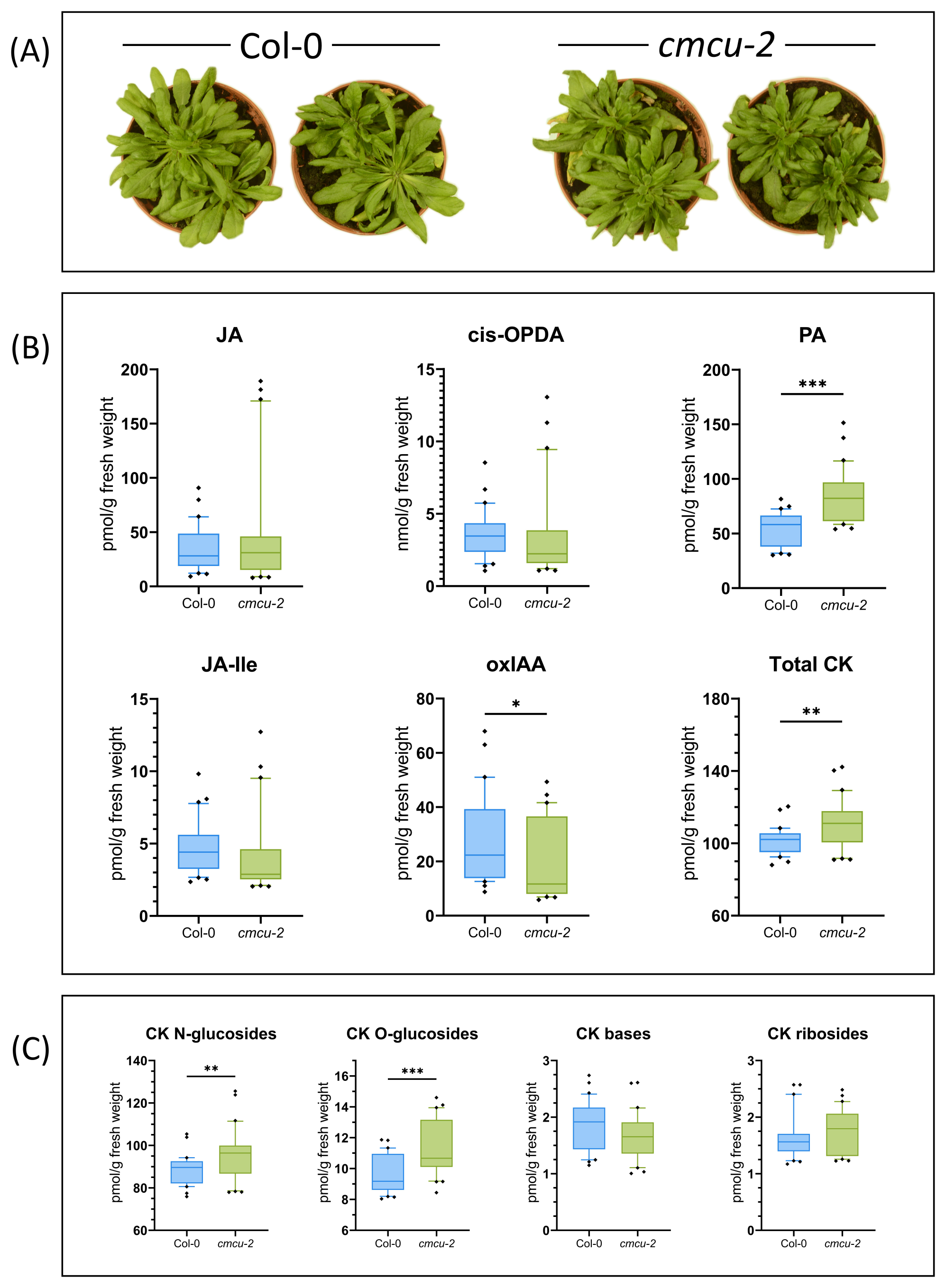

Supplement: Supplementary Figure 2 — Additional results of hormone quantifications (n=30). (A) Col-0 and cmcu-2 plants sampled for hormone quantifications. (B) Box plots of hormone abundance for molecules in the jasmonate (JA, cis-OPDA and Ja-Ile), ABA (ABA, PA) and auxin (IAA, oxIAA) families, together with total cytokinins abundance (n=30). (C) Box plots of hormone abundance for molecules in the cytokinin family. [file Image_2.tif]

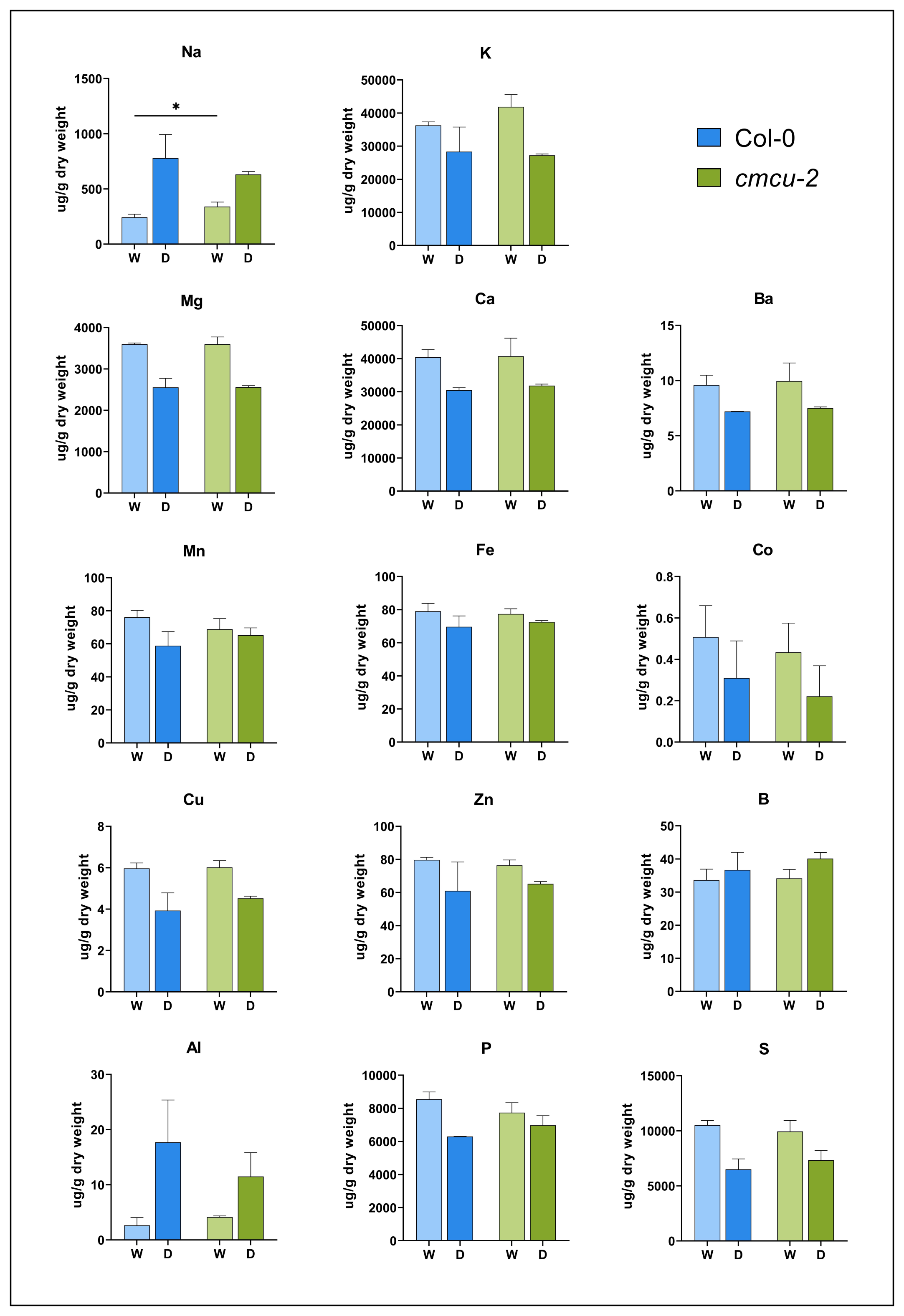

Supplement: Supplementary Figure 3 — Ionomic analysis of Col-0 and cmcu-2 non-stressed (watered, W, n=3) and drought-stressed (D, n=2) plants. Element concentration in plants rosettes was normalized on plant tissue dry weight, and reported as mean + SD. [file Image_3.tif]

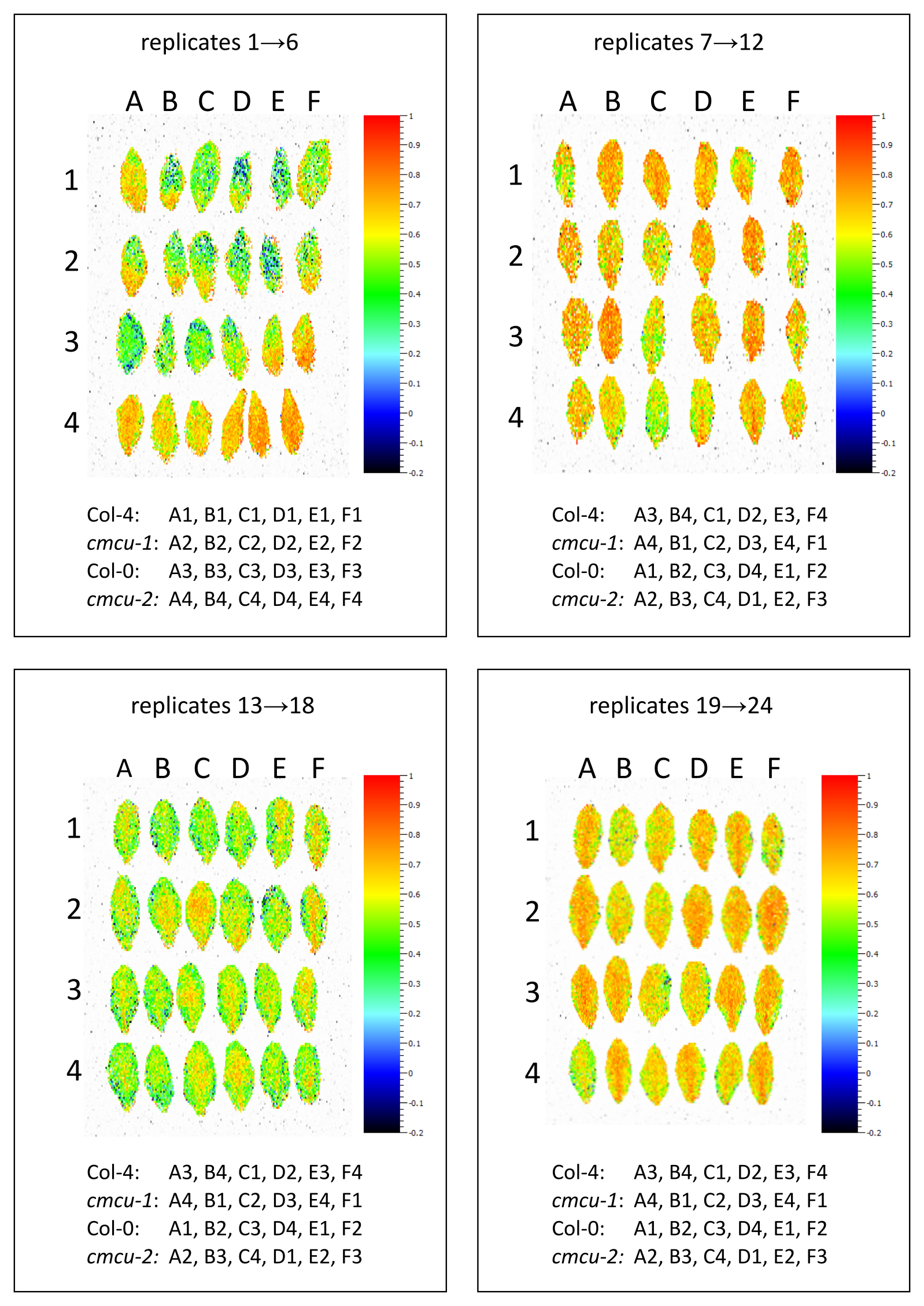

Supplement: Supplementary Figure 4 — Leaf images of Fv/Fm for all replicates (n=24) shown in the photoinhibition experiment shown in , after 24 hours of high light treatment at 1500 photons µmol m-2 s-1. Leaf genotype is indicated in the table below images. [file Image_4.tif]

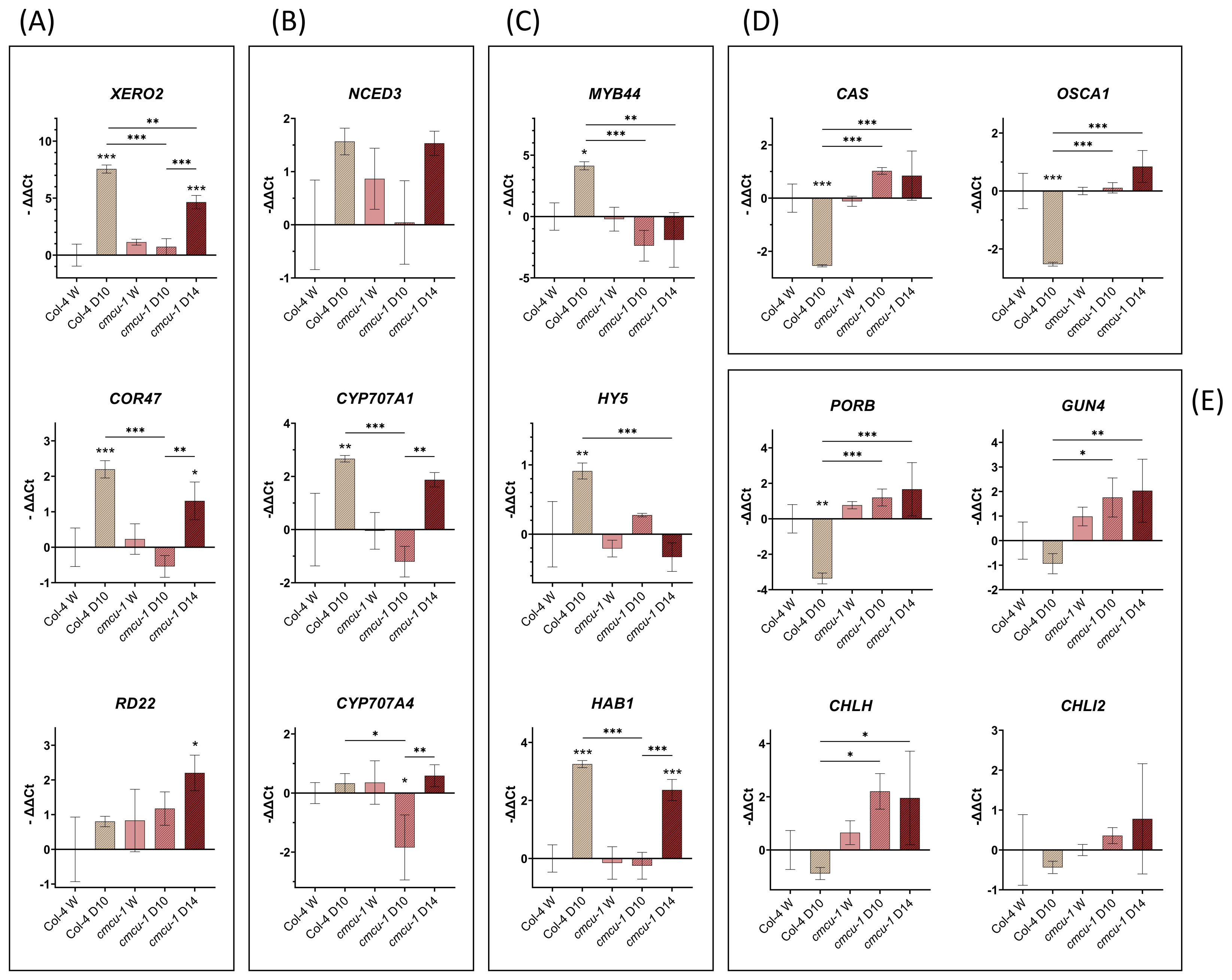

Supplement: Supplementary Figure 5 — RT-qPCR expression results for genes of interest in cmcu-1 and Col-4 watered and drought-stressed samples. Transcript level of drought marker genes (A), genes involved in ABA de novo biosynthesis and degradation (B), other genes of interest involved in stress response (C, D) and genes encoding chlorophyll biosynthesis pathway proteins (E). Expression levels are reported as -ΔΔCt (mean ± SD, n=3) for Col-4 WT and cmcu-1 leaves in watered (marked as W) and drought stress conditions, lasting either 10 or 14 days (marked as D10 and D14). Asterisks on bars are used to highlight significant differences of the sample with respect to the control (Col-4 watered sample), while asterisks on horizontal lines highlight significant differences in other comparisons. [file Image_5.png]

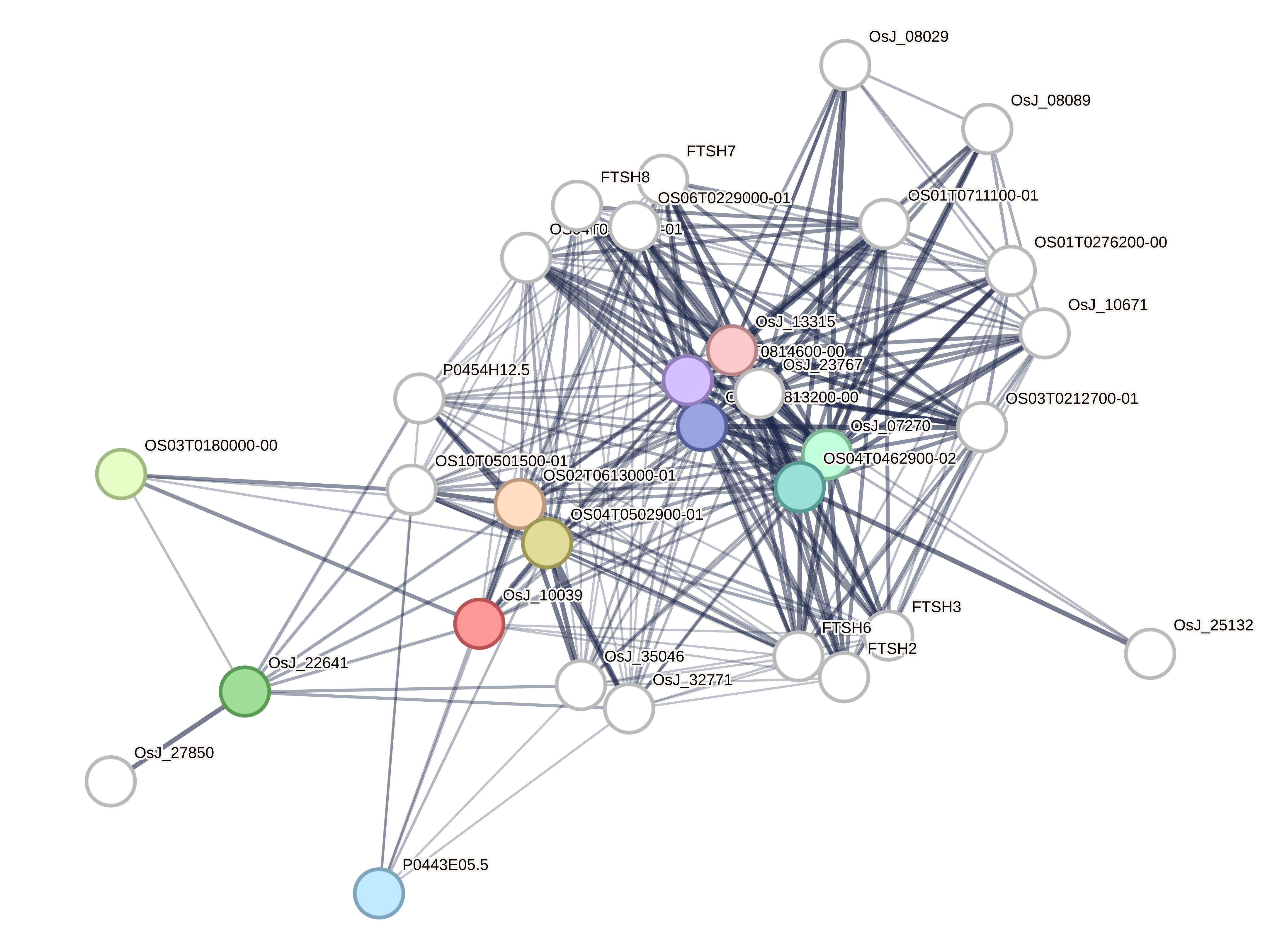

Supplement: Supplementary Figure 6 — Putative interactor network of MCU homolog in Oryza sativa OS10T0501500-01 according to STRING interaction database. [file Image_6.png]
