# Supplementary material for: Comparative analysis of wild-type and chloroplast MCU-deficient plants reveals multiple consequences of chloroplast calcium handling under drought stress
Source: Front Plant Sci. 2023 Aug 25;14:1228060. doi: 10.3389/fpls.2023.1228060 (PMC10485843; doi:10.3389/fpls.2023.1228060)
Supplement: Supplementary file 8 [file Table_2.docx]

|  |  |  | **number of proteins** | | 5 | 19 | 22 | 34 | 11 | 21 |
| --- | --- | --- | --- | --- | --- | --- | --- | --- | --- | --- |
|  |  |  | **fold enrichment** | | 19,07 | 3,89 | 3,88 | 2,84 | 4,78 | 2,24 |
|  |  |  | **FDR** | | 4,3E-03 | 1,3E-04 | 1,9E-05 | 3,6E-06 | 8,38E-03 | 8,07E-02 |
|  |  |  | |  | **GO:0009627** | **GO:0098542** | **GO:0009607** | **GO:0006950** | **GO:0009414** | **GO:0009628** |
| **gene id** | **protein** | **logFC** | **direction** | **hit annotation** | systemic acquired resistance | defense response to other organism | response to biotic stimulus | response to stress | response to water deprivation | response to abiotic stimulus |
| At3g57260 | BG2 | 1,53 | **up** | hit | 5 | 6 | 7 | 11 |  | 9 |
| At3g33530 | At3g33530 | 1,47 | **up** | hit |  |  |  |  |  |  |
| At5g10760 | AED1 | 1,14 | **up** | hit | 2 | 8 | 10 | 26 |  |  |
| At4g25000 | AMY1 | 1,07 | **up** | hit |  |  |  |  |  |  |
| At3g09940 | MDAR3 | 0,98 | **up** | hit |  |  | 8 | 12 | 11 | 10 |
| At2g39420 | At2g39420 | 0,67 | **up** | hit |  | 5 | 6 | 7 | 8 | 6 |
| At1g14880 | PCR1 | 0,66 | **up** | hit |  | 2 | 2 | 22 |  |  |
| At1g08450 | CRT3 | 0,64 | **up** | hit |  | 3 | 3 | 3 |  |  |
| At2g31865 | PARG2 | 0,62 | **up** | hit |  | 16 | 18 | 18 |  |  |
| At2g30140 | UGT87A2 | 0,57 | **up** | candidate |  |  |  |  |  |  |
| At3g51450 | SSL7 | 0,57 | **up** | candidate |  |  | 5 | 24 |  |  |
| At5g26340 | STP13 | 0,56 | **up** | candidate |  |  |  | 8 | 9 | 7 |
| At4g05020 | NDB2 | 0,55 | **up** | candidate |  |  |  |  |  |  |
| At2g18660 | EGC2 | 0,54 | **up** | candidate | 1 | 7 | 9 | 13 |  |  |
| At4g36945 | At4g36945 | 0,51 | **up** | candidate |  | 4 | 4 | 23 |  |  |
| At3g19010 | At3g19010 | 0,51 | **up** | candidate |  | 12 | 14 | 29 | 4 | 14 |
| At1g07990 | At1g07990 | 0,50 | **up** | candidate |  |  |  |  |  |  |
| At1g77510 | PDIL1-2 | 0,49 | **up** | candidate |  |  |  | 31 |  | 16 |
| At2g15390 | FUT4 | 0,49 | **up** | candidate |  |  |  | 25 |  | 12 |
| At1g06760 | At1g06760 | 0,47 | **up** | candidate |  |  |  |  |  |  |
| At4g24190 | HSP90-7 | 0,47 | **up** | candidate |  |  |  | 6 | 7 | 5 |
| At1g45145 | TRX5 | 0,46 | **up** | candidate |  | 18 | 21 | 33 |  |  |
| At1g72330 | ALAAT2 | 0,45 | **up** | candidate |  |  |  | 16 |  | 17 |
| At3g57280 | FAX1 | 0,44 | **up** | candidate |  |  |  |  |  |  |
| At5g37600 | GLN1-1 | 0,43 | **up** | candidate |  |  |  |  |  |  |
| At1g48320 | DHNAT1 | 0,43 | **up** | candidate |  |  |  |  |  |  |
| At5g03350 | LLP | 0,41 | **up** | candidate | 3 | 10 | 12 | 28 |  |  |
| At2g25110 | SDF2 | 0,40 | **up** | candidate |  | 17 | 19 | 19 |  |  |
| At4g39090 | RD19A | 0,39 | **up** | candidate |  | 1 | 1 | 1 | 1 | 1 |
| At3g20250 | APUM5 | 0,38 | **up** | candidate |  | 11 | 13 | 14 | 3 | 13 |
| At2g22500 | PUMP5 | 0,38 | **up** | candidate |  |  |  | 15 |  | 15 |
| At2g29720 | CTF2B | 0,38 | **up** | candidate | 4 | 14 | 16 | 32 | 5 | 18 |
| At4g29380 | VPS15 | 0,37 | **up** | candidate |  |  |  |  |  |  |
| At3g24190 | At3g24190 | -0,30 | **down** | candidate |  |  |  | 20 | 10 | 20 |
| At1g01320 | REC1 | -0,30 | **down** | candidate |  |  |  |  |  |  |
| At4g39540 | SK2 | -0,31 | **down** | candidate |  |  |  |  |  |  |
| At4g18740 | At4g18740 | -0,33 | **down** | candidate |  |  |  |  |  | 19 |
| At2g31890 | RAP | -0,34 | **down** | candidate |  |  |  |  |  |  |
| At3g57320 | F28O9.170 | -0,35 | **down** | candidate |  |  |  |  |  |  |
| At3g04550 | RAF1.2 | -0,35 | **down** | candidate |  |  |  |  |  |  |
| At4g34260 | FUC95A | -0,37 | **down** | candidate |  | 9 | 11 | 27 |  |  |
| At2g22990 | SCPL8 | -0,37 | **down** | candidate |  |  |  |  |  |  |
| At1g58290 | HEMA1 | -0,38 | **down** | candidate |  |  |  |  |  | 21 |
| At5g08610 | RH26 | -0,39 | **down** | candidate |  |  |  |  |  |  |
| At3g27960 | KLCR2 | -0,41 | **down** | candidate |  |  |  |  |  |  |
| At2g39140 | SVR1 | -0,41 | **down** | candidate |  |  |  |  |  |  |
| At1g65590 | HEXO3 | -0,42 | **down** | candidate |  |  |  |  |  |  |
| At3g06430 | EMB2750 | -0,42 | **down** | candidate |  |  |  |  |  |  |
| At1g76160 | sks5 | -0,43 | **down** | candidate |  | 19 | 22 | 34 |  |  |
| At1g73080 | PEPR1 | -0,43 | **down** | candidate |  | 15 | 17 | 17 |  |  |
| At4g22290 | At4g22290 | -0,44 | **down** | candidate |  |  |  |  |  |  |
| At4g23400 | PIP1-5 | -0,47 | **down** | candidate |  |  |  | 4 | 2 | 3 |
| At2g29630 | THIC | -0,50 | **down** | candidate |  |  | 20 |  |  |  |
| At3g13790 | CWINV1 | -0,50 | **down** | candidate |  | 13 | 15 | 30 |  |  |
| At5g13650 | SVR3 | -0,52 | **down** | candidate |  |  |  | 9 |  | 8 |
| At5g26742 | RH3 | -0,52 | **down** | candidate |  |  |  | 2 |  | 2 |
| At2g39670 | At2g39670 | -0,53 | **down** | candidate |  |  |  |  |  | 11 |
| At3g59040 | At3g59040 | -0,60 | **down** | hit |  |  |  |  |  |  |
| At4g36390 | At4g36390 | -0,62 | **down** | hit |  |  |  |  |  |  |
| At3g21670 | NPF6.4 | -0,65 | **down** | hit |  |  |  | 5 | 6 | 4 |
| At3g06980 | RH50 | -0,69 | **down** | hit |  |  |  | 21 |  |  |
| At3g53260 | PAL2 | -0,73 | **down** | hit |  |  |  | 10 |  |  |
| At1g31690 | At1g31690 | -0,78 | **down** | hit |  |  |  |  |  |  |
