# Supplementary material for: Comparative analysis of wild-type and chloroplast MCU-deficient plants reveals multiple consequences of chloroplast calcium handling under drought stress
Source: Front Plant Sci. 2023 Aug 25;14:1228060. doi: 10.3389/fpls.2023.1228060 (PMC10485843; doi:10.3389/fpls.2023.1228060)
Supplement: Supplementary file 10 [file Table_4.docx]

| ***cmcu-1*** |  |  |  |  |
| --- | --- | --- | --- | --- |
| **Gene name** | **logFC** | **p-value** | **FDR** | **Hit annotation** |
| COR47 | 1,829 | 6,80E-06 | 9,55E-04 | hit |
| XERO2 | 4,013 | 4,40E-04 | 8,49E-03 | hit |
| ERD10 | 2,118 | 3,25E-06 | 7,05E-04 | hit |
| ERD14 | 1,149 | 7,43E-03 | 5,01E-02 | candidate |
| RAB18 | 2,386 | 1,65E-04 | 4,65E-03 | hit |
| RD22 | 0,810 | 6,27E-04 | 1,08E-02 | candidate |
|  |  |  |  |  |
| ***cmcu-2*** |  |  |  |  |
| **Gene name** | **logFC** | **p-value** | **FDR** | **Hit annotation** |
| COR47 | 3,736 | 1,37E-07 | 3,11E-04 | hit |
| XERO2 | 2,370 | 5,54E-03 | 1,41E-02 | hit |
| ERD10 | 2,221 | 2,51E-06 | 4,46E-04 | hit |
| ERD14 | 0,762 | 3,71E-02 | 6,30E-02 | candidate |
| RAB18 | 2,625 | 1,00E-04 | 1,43E-03 | hit |
| RD22 | 0,744 | 9,61E-04 | 4,35E-03 | candidate |
